# Principles governing control of aggregation and dispersion of aqueous graphene oxide

**DOI:** 10.1038/s41598-021-01626-3

**Published:** 2021-11-17

**Authors:** James L. Suter, Peter V. Coveney

**Affiliations:** 1 Centre for Computational Science, University College London, 20 Gordon Street, London, WC1H 0AJ UK; 2 Computational Science Laboratory, Institute for Informatics, Faculty of Science, University of Amsterdam, 1098XH Amsterdam, The Netherlands

**Keywords:** Nanoscale materials, Theory and computation, Computational chemistry

## Abstract

Controlling the structure of graphene oxide (GO) phases and their smaller analogues, graphene (oxide) quantum dots (GOQDs), is vitally important for any of their widespread intended applications: highly ordered arrangements of nanoparticles for thin-film or membrane applications of GO, dispersed nanoparticles for composite materials and three-dimensional porous arrangements for hydrogels. In aqueous environments, it is not only the chemical composition of the GO flakes that determines their morphologies; external factors such as pH and the coexisting cations also influence the structures formed. By using accurate models of GO that capture the heterogeneity of surface oxidation and very large-scale coarse-grained molecular dynamics that can simulate the behaviour of GO at realistic sizes of GOQDs, the driving forces that lead to the various morphologies in aqueous solution are resolved. We find the morphologies are determined by a complex interplay between electrostatic, ${\pi }$–${\pi }$ and hydrogen bonding interactions. Assembled morphologies can be controlled by changing the degree of oxidation and the pH. In acidic aqueous solution, the GO flakes vary from fully aggregated over graphitic domains to partial aggregation via hydrogen bonding between hydroxylated domains, leading to the formation of planar extended flakes at high oxidation ratios and stacks at low oxidation ratios. At high pH, where the edge carboxylic acid groups are deprotonated, electrostatic repulsion leads to more dispersion, but a variety of aggregation behaviour is surprisingly still observed: over graphitic regions, via hydrogen bonding and “face-edge” interactions. Calcium ions cause additional aggregation, with a greater number of “face-face” and “edge-edge” aggregation mechanisms, leading to irregular aggregated structures. “Face-face” aggregation mechanisms are enhanced by the GO flakes possessing distinct domains of hydroxylated and graphitic regions, with ${\pi }$–${\pi }$ and hydrogen bonding interactions prevalent between these regions on aggregated flakes respectively. These findings furnish explanations for the aggregation characteristics of GO and GOQDs, and provide computational methods to design directed synthesis routes for self-assembled and associated applications.

## Introduction

Due to the unique hydrophilic, thermal and electrical properties of GO, there is much interest in using the chemistry of GO flakes to create self-assembled GO systems, with their morphologies targeted to applications^1^. Films and fibres require high orientation and strong interactions between the flakes to increase the mechanical strength and modulus^2,3^. However, for energy devices, such as supercapacitors and hydrogels, porous structures are desired^4,5^. For some applications, dispersion is required; aggregation and the associated reduction in surface area has been reported as a predominant factor in decreasing the protein-binding capacities of GO, which has implications in the pharmaceutical applications of graphene-based materials^6^. Similarly, GO is seen a potential adsorbent for the removal of environmental contaminants, for example through the formation of nanoparticulate aggregates containing radionuclides^7^.

Recently, there has been much interest in Graphene-Oxide Quantum Dots (GOQDs), which can be viewed as a scaled down version of GO^8,9^. The typical size of a GOQD flake is between 2–20 nm^10^, compared to typical diameters of micrometers for GO. Due to edge and quantum confinement effects, GOQDs have shown potential applications in bioimaging, bio- and metal sensing, photovoltaics and photocatalysts. Due to the greater proportion of edge sites compared to GO, GOQDs have greater hydrophilicity while continuing to possess the same amphiphilic properties as GO^10^. One important application of GOQDs is in photoluminescence (PL)-related fields. The photoluminescence (PL) spectra of the GOQDs in self- assembled and fully exfoliated forms have been shown to be completely different^11–13^, due to aggregation-mediated energy level reconstruction.

Experimentally, it has been observed in ultrahigh-resolution transmission electron microscopy (TEM) images that both GO and GOQDs contain domains of oxidation and graphitic regions, rather than a homogeneous distribution of oxidation sites across the GO surface^14^. These regions can become the building blocks that self-assemble into desired 3D architectures, or be the reason for the formation of unwanted aggregated structures. Therefore, control of secondary interactions such as electrostatic, $\pi$–$\pi$ and hydrogen bonding is required, either by altering the chemical structure of the flakes or through changing external factors, such as pH or counterions present^13^. In this paper we explore the aggregation modes of GO and GOQD flakes in different aqueous environments to determine the roles of these different interactions. To do so experimentally is challenging due to difficulties in finely characterising GO. GO can vary significantly depending on the method used to create it and the extent of oxidation, leading to a wide distribution of flake sizes and composition of functional groups, depending on the production method^15^.

Experimentally, several factors have been shown to affect the aggregation of GO in water. It is well known that GO is highly soluble in water, where the large number of oxygen functional groups are deprotonated^16^. At low pH, the oxygen functional groups are protonated and the surface charge density of GO is reduced. With the reduction of electrostatic repulsion, large-scale visible flocculent aggregation occurs^17,18^. Increasing the ionic strength of the aqueous solution screens the negative charge and the critical coagulation concentration is reduced. Both these properties have been explored using Derjaguin–Landau–Verwey–Overbeek (DLVO) theory in aqueous media by Chowdhury et al.^19^ and Gudarzi^20^, who modified the Hamaker constants and van der Waals forces to account for the 2-dimensional shape of GO flakes. Both studies showed that the collodial stability is due to electrostatic repulsion, which overcomes the attractive van der Waals forces.

However, while DLVO theory captures the general behaviour of GO in aqueous solution, GO shows unexpected behaviour in organic solvents: GO is soluble in DMF, *N*-methyl-2-pyrrolidone and ethylene glycol but insoluble in methanol and ethanol^21^, despite the ability of the latter two solvents to form hydrogen bonds with the oxygen functional groups of GO. Indeed, GO can be transferred into the alcohol phase by gradual replacement of the water molecules. Neklyodov explained these observations through modelling, which showed the interactions between the hydroxyl groups and solvent molecules are the key determinant of solubility; strong hydrogen bonds between alcohol groups lead to solubility in water, but the alkyl groups of methanol and ethanol result in a hydrophobic shell around GO^22^. These results show that the chemical interactions between the GO functional groups and solvent molecules can be the driver for aggregation behaviour, and can be missed when using mean-field theories or looking at macroscopic quantities, such as comparing Hildebrand parameters. Other studies have also shown GO behaviour that is not fully explained by DLVO theory. Qi et al. showed that, in the presence of ions with small hydration radii, it is the type and number of oxygen functional groups that affects the aggregation tendency, not necessarily the degree of oxidation^23^. Dimiev and co-workers used the NMR relaxation method to investigation the interactions between GO and metal cations^24^. They found that the interaction between transition metal cations and GO are chemical in nature. The metals formed coordinate-complex bonds between the oxygen functional groups, replacing water molecules^25,26^. These bound ions can be viewed as the Stern-layer, as opposed to electrostatically attracted ions which form a diffuse layer^27^. Several studies have examined the aggregation of GO in the presence of calcium chloride, which have shown that the aggregation of GO is related to the adsorption of calcium on the functional groups of GO, including the ability to form bridges between two flakes^18,28^.

It is clear that the dispersion or assembly of GO flakes in aqueous solution is the result of a complex interaction between surface properties and the chemistry of the solution. Due to the variability of structure and functional groups depending on the experimental method of creating GO, molecular simulation is an ideal approach to ascertain how the chemistry on the surfaces and edges of aqueous GO and GOQDs flakes, and their interaction with the aqueous solution, is correlated with their aggregation behaviour.

As we reported in our previous study investigating the structures formed by GO flakes in polymeric media^29^, molecular simulation is a powerful method by which to study these systems because we can control the composition and structure of GO, examine thermodynamically stable morphologies of GO and view dispersion and aggregation mechanisms with a high level of fidelity. We can observe whether the aggregation modes observed in our simulations are those that have been proposed, such as edge-to-edge and face-to-face binding^18,30^. By examining in atomistic detail the interactions present in our aggregated GO flakes, we can observe the balance between interactions with the water molecules, ions and other flakes. Ultimately, our aim is to provide computational methods to design synthesis routes for different GO and GOQD self-assemblies^31^ and applications^32^ in aqueous solution.

We have recently introduced an algorithm that uses the reactivity of oxidised intermediates to create accurate models of GO, which captures the two-phase nature of the GO surface^33^ (for more details, see Computational Methods and Supporting Information). As we show in this paper, these localised domains of oxidation on the GO flake surface are important for determining the aggregated morphology. In this study, we combine our accurate models of GO surfaces with multiscale modelling, comprising coarse-grained (CG) and atomistic molecular dynamics. CG simulations can approach length and time scales of microns and microseconds respectively, while still retaining atomistic information, as demonstrated by our previous study of self-assembly of GO in polymer melts. Such lengths and timescales, commonly referred to as the meso-scale, are required to observe the self-assembly and dispersion processes^29,34,35^. Through the use of high-performance computing resources and coarse-grained molecular dynamics, we can access length scales where GOQDs can be simulated at both at realistic sizes and low volume fractions (approximately < 3.5 %), while retaining atomistic resolution. We also access timescales which are long enough to observe the evolution from dispersed GOQD flakes to aggregated morphologies. As the percentage of edge sites compared to basal surface sites is small in our models (< 5%), we can assume the results of our simulations will be also relevant to those for GO, as well as directly comparable to experimental observations of aqueous GOQDs. Here, we examine the effects of chemical composition (flake oxygen content, the distribution of oxygen and the protonation state of the flake edge groups) on the properties of GO in aqueous solution. Aqueous solution is the ideal medium for GO processing compared to other organic solvents used for liquid phase exfoliation due to their toxicity and incompatibility with other aspects of processing and the solubility of GO in water. Our results provide unique insights into the 3D self-assembly mechanisms and the associated interactions in water, including illustrating how the surface morphology and edge oxygen-containing groups can be modified to realise controllable characteristics.

## Computational methods

Graphene oxide has functional groups on the basal plane and the free edges. To simplify our coarse-grained GO models, we only consider oxidation on the basal plane in the form of hydroxyl groups in order to maintain focus on the comparison of oxidation level without variation in oxidation type. All of the models contain carboxylic acid groups on the edges of the flakes. These groups are either protonated or unprotonated. 25% of the edge carbon atoms are replaced by carboxoxylic acids. For unprotonated, i.e. charged, carboxylic acid groups, a charge balancing counterion was added to the system next to the functional group. Two counterions were chosen: $\hbox {Na}^{+}$ and $\hbox {Ca}^{2+}$. The protonated neutral carboxylic acid groups correspond to low pH conditions, less than the $\hbox {pK}_{{a}}$ of 4.3 for ionization of the carboxylic acid group^36^.

**Figure 1:**
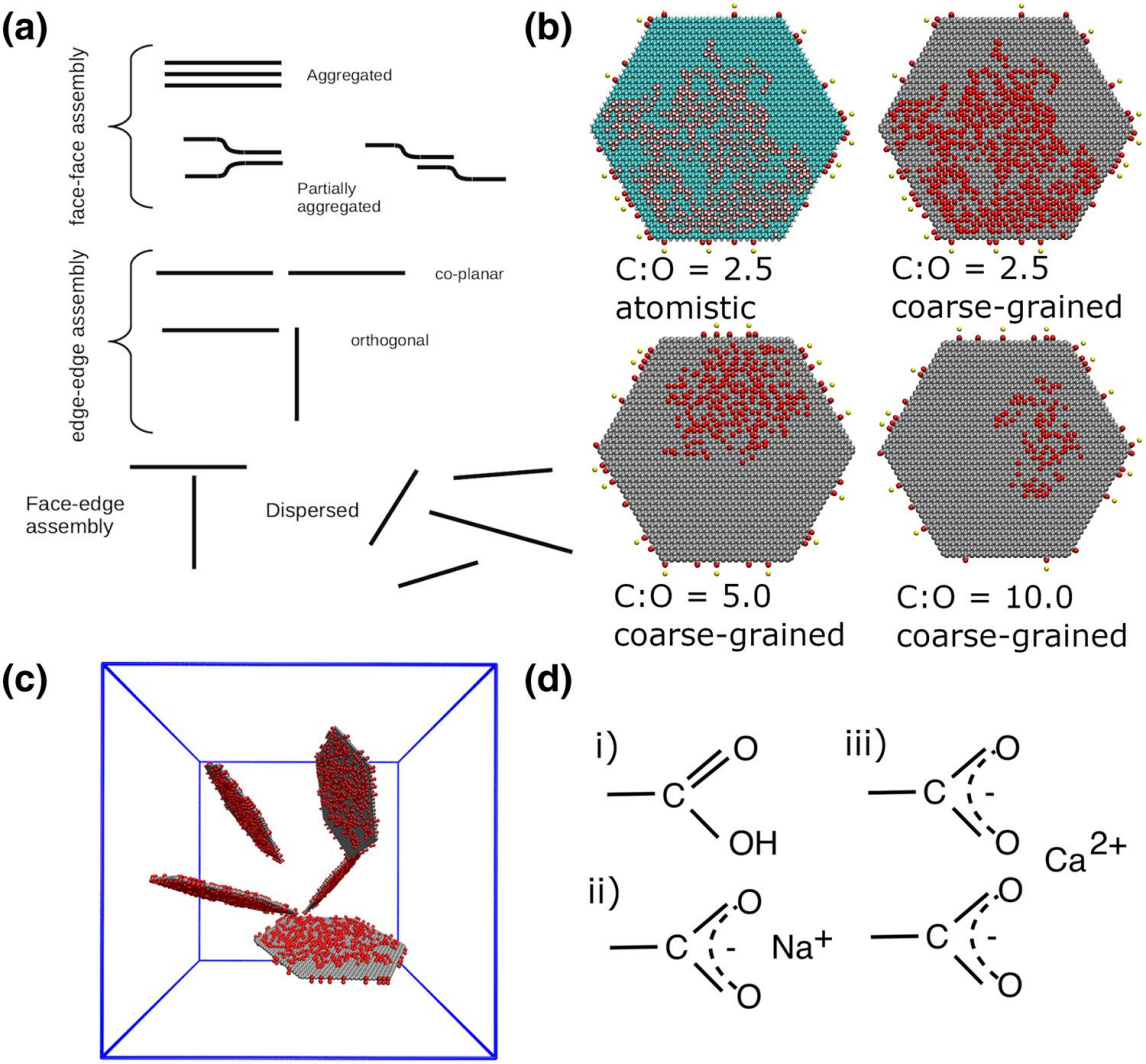
(**a**) Schematic diagram displaying the different morphologies and aggregation interaction modes formed by the graphene (oxide) flakes. The flakes at the top right (**b**) are examples of GO sheets oxidised to different extents. The first flake shows the GO flake with a C:O ratio of 2.5 at the atomistic level. In this example, the carboxylic edge groups are deprotonated with a calcium counterion. The colour scheme for atomistic systems is: carbon is cyan, oxygen is red, hydrogen is white, calcium is yellow and sodium is blue. The remaining flakes are shown at the coarse-grained level. The red circles represent the oxidised hydroxyl groups and edge carboxylic groups, and grey represents graphitic carbon atoms. The diameter of each flake is 10 nm. (**c**) shows the dispersed initial configuration (water not shown). There are five flakes within each simulation cell. The solid blue lines are the periodic boundaries, with lattice parameters of approximately 18nm. d) shows the different carboxylic edge groups considered in this study, (i) prontonated \ neutral (i.e low pH), (ii) deprotonated with sodium counterions and (iii) deprotonated with calcium counterions. All atomistic and coarse-grained visualisations in this and subsequent figures were created using VMD Version 1.9.2^48,49^.

To generate the distribution of oxidation sites on the basal surfaces of the GO flake, we used our recently developed algorithm, which employs quantum mechanical simulations of reactive intermediates^37^ to determine the progress of oxidation on the flake surface^33^. In contrast to a random distribution of oxidation sites over the surface, based on the the Lerf-Klinowski model^38^, our algorithm creates GO structures with distinct oxidised and graphene domains, as visualised in experiments. Figure 1 shows the snapshots from our simulations, which illustrate the distinct domains of *sp*$^{2}$ and *sp*$^{3}$ phases as created by our GO model builder.

*Coarse-grained potentials* Water molecules in our coarse-grained molecular dynamics simulations are represented by the mW potential^39^, where each water molecule is represented by a single coarse-grained particle that interacts via the Stillinger–Weber (SW) potential, which contains both 2- and 3-body terms^40^. The short range nature of the potential, combined with a reduction in the number of atoms, results in a considerable reduction in computational cost compared to full atomistic simulations of water. To create the interactions between GO and mW water, we built upon the work of Islam et al., who generated coarse-grained potentials based on mW water for protonated and deprotonated carboxylic acids, which were applied to poly(methacrylic acid) (PMAA)^41^. These potentials were used to represent the interactions between the carboxylic acid groups and mW water. A list of the potential terms is given in Supporting Information Table S1.

The GO flakes are represented by a unified-atom representation. The *sp*${^2}$ carbon atoms of the GO framework have a 1:1 mapping to CG atoms, and interact with other *sp*${^2}$ carbon atoms using the OPLS potential^42^, in the same manner as atomistic graphene (called CG type C). The edge of the graphene flakes is represented by an united atom type which maps the edge carbon and hydrogen atoms into a single CG bead (type CH); the hydroxyl atoms (O and H) on the GO basal surface are represented by a single CG atom (type OH). As per Islam et al., the carboxylic acid groups are represented by a three CG atoms, corresponding to the $\alpha$ carbon and the oxygen atoms^41^. Interaction potentials not defined by either the OPLS forcefield or the potential set of Islam et al. are computed using the iterative Boltzmann inversion (IBI) scheme to create a tabular potential. These include interactions between ions ($\hbox {Ca}^{2+}$, $\hbox {Na}^{+}$ and $\hbox {COO}^{-}$). To create the interaction potentials between the mW water atoms and the graphene surface, we constructed interaction potentials that recreate the atomistic density profiles perpendicular for the graphene surface, calculated from fully atomistic simulations of the graphene-water interface using the OPLS forcefield and TIP4P/Ew water^43^. All non-bonded potentials in the GO flake (bonds, angles, dihedrals and impropers) use the OPLS forcefield parameters. See Supporting Information Sections S1–S2 for more details. The set of atomistic simulations were performed using the OPLS forcefield^42^. Underpinning everything, the work described in this article is based on the GraFF forcefield which we introduced for graphene three years ago^44^.

*Coarse-grained model systems* The GO water system was created by randomly placing five hexagonal flakes of 10 nm diameter into a simulation cell of dimensions 20 nm $\times$ 20 nm $\times$ 20 nm. Water molecules were then added around the flakes with a density of 0.7 g/mL. Each flake is comprised of between 2900 and 4500 atoms, depending on the degree of oxidation. The atomistic systems were subsequently relaxed for 2 ns using *NpT* conditions at 300 K and mapped to their CG equivalents. The CG simulations were performed in the *NVT* ensemble, with the relaxed atomistic lattice parameters of between 17.9 nm and 18.1 $\hbox {nm}^{3}$. The procedure is described in Supporting Information Section S3 and the different systems are listed in Supporting Information Table S2.

To reach thermodynamic equilibrium, we used a simulated annealing approach to overcome energy barriers. The simulations were heated up to 500 K and run at this temperature for at least 100 ns, and cooled down to 300 K over 30 ns. The simulated annealing approach is used to find energetically favorable minima at 300 K. No bond breaking or formation is possible in the classical atomistic and coarse-grained molecular dynamics simulations, therefore no reactions are simulated at higher temperatures; instead configurational space is explored before gradually cooling down to 300 K. Due to the softness of the coarse-grained potentials, the dynamics are much faster. Subsequent analysis on the structures formed by the GO/water systems is performed at 300 K.

*Ensembles and averages* To increase our sampling, we created 9 replicas of each system, from 3 randomly dispersed configurations. These replicas were given a different random seed for the generation of atomic velocities drawn from a Maxwell-Boltzmann distribution. All simulations were performed using the LAMMPS molecular dynamics code^45,46^. Replica simulation building and subsequent analysis used the VECMA toolkit^47^ (www.vecma-toolkit.eu/toolkit).

## Results and discussion

We consider three different oxidation states of GO flakes, with varying C:O atomic ratios of 10.0, 5.0 and 2.5. Typical values of GO oxidation found experimentally lie between 4 and 2; reduced graphene-oxide or modified oxidation methods with reducing reagents have ratios greater than 4^50,51^. GO flakes of diameter 10 nm were chosen. Although this is small compared to typical experimental sizes of GO, it is within the size ranged reported for Graphene-Oxide Quantum Dots (GOQDs) of between 2–20 nm^10^. In the study of Le et al., the average particle size of GOQDs produced was 9.0 nm^30^.

On the edges of the GO flake, we converted 25% of the edge graphene atoms to carboxylic acid groups. To correspond to low pH, these groups were in their uncharged, protonated form. To correspond to high pH, the groups were in their charged, deprotonated form. We have simulated two different counterions which neutralise the charged carboxylic acid groups: $\hbox {Na}^{+}$ and $\hbox {Ca}^{2+}$.

Each coarse-grained simulation contains five hexagonal GO flakes with a volume fraction of approximately 3.5%. Each system is therefore very large-scale; including water molecules, they are composed of approximately 590,000 atoms at atomistic resolution and 205,000 CG atoms at coarse-grained resolution. For the purposes of sampling and uncertainty quantification, we use ensembles of simulations for each oxidation state consisting of three replicas starting from different randomly dispersed configurations, of which each had three replicas created, forming an ensemble of nine replicas in total (see Computational Methods and Supporting Information). In total, across the 9 different oxidation states and edge groups, 81 simulations have been performed, allowing us to create statistical distributions of the aggregated structures formed. The flakes and initial structures are shown in Fig. 1.

Each replica contains a different initial set of velocities drawn randomly from a Maxwell–Boltzmann distribution, and is simulated using a simulated annealing routine by heating to a high temperature (500K) followed by cooling down to room temperature (see Computational methods). This procedure ensures as far as possible that we probe thermodynamic equilibrium properties^52^.

To further examine the structures formed with atomistic resolution, we selected three GO models to be simulated at the atomistic level using the OPLS forcefield (for more details see Computational Methods). Due to the much higher computational cost, the atomistic models were run for 30 ns at 500 K followed by cooling down to room temperature. This timescale, while much reduced compared to CG equivalents, was long enough to observe several of the same binding motifs seen in the CG simulations, allowing us to perform analysis in full atomistic resolution. The models selected have a C:O atomic ratio of 2.5, and were simulated at low pH (protonated edge groups) and a high pH (charged edge groups) with $\hbox {Na}^{+}$ and $\hbox {Ca}^{2+}$ ions respectively.

**Figure 2:**
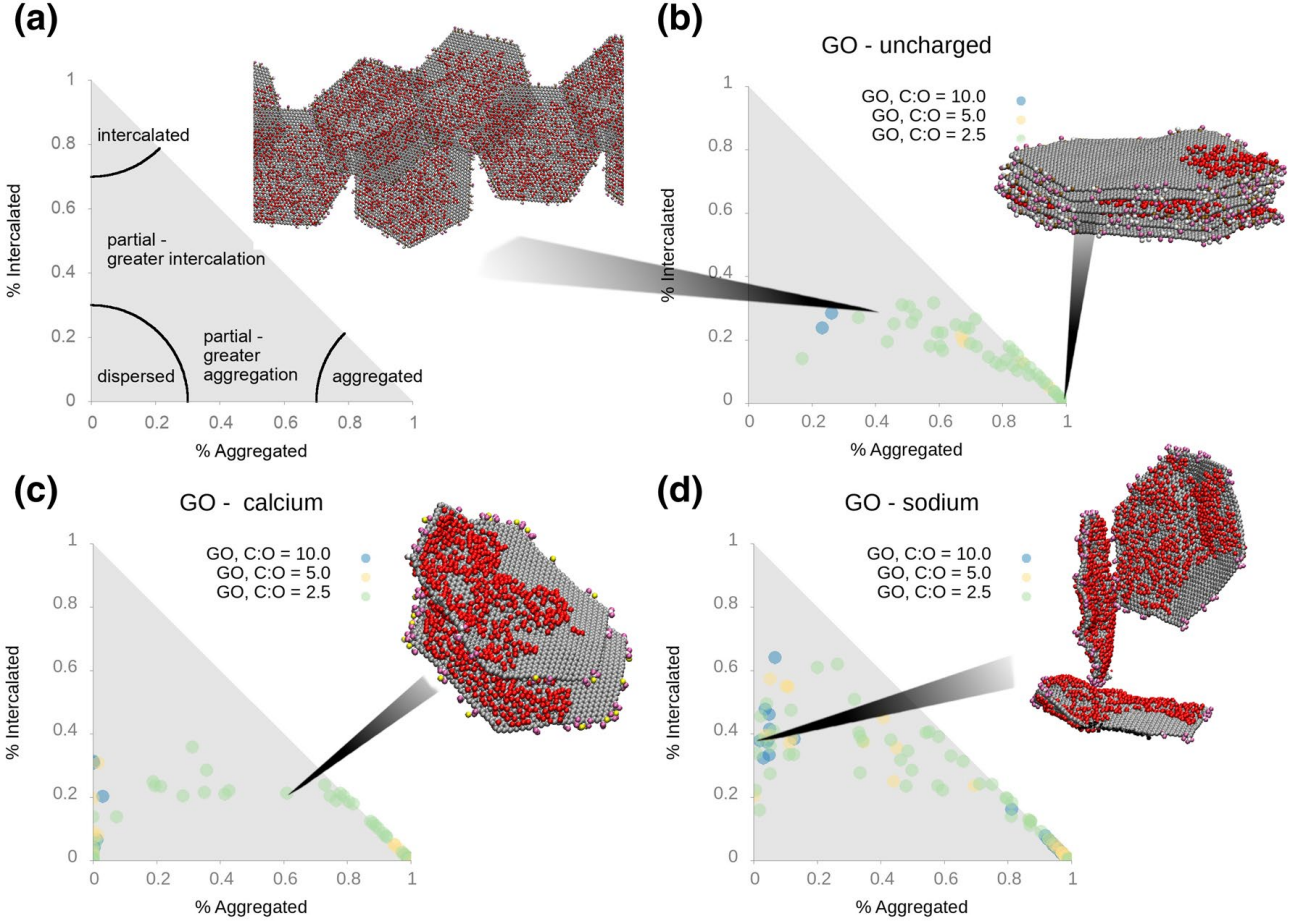
The final morphologies of graphene oxide flakes simulated in aqueous solution. Panel (**a**) illustrates our morphology classification as a function of % of atoms in aggregated and dispersed environments. (**b**) corresponds to protonated carboxylic acid groups on the edge of the graphene flakes, i.e. low pH; (**c**) corresponds to unprotonated (charged) carboxylic acid groups with $\hbox {Ca}^{2+}$ counterions; (**d**) corresponds to unprotonated (charged) carboxylic acid groups with $\hbox {Na}^{+}$ counterions. Each point represents a flake: on each graph there are 3 different oxidation states, each with 9 replica simulations comprising 5 flakes, resulting in a total of 135 points. The average values for each system are listed in Supporting Information Table S3.

To analyse the morphologies found in our CG simulations, we categorise them in a similar manner to those of our previous study of GO/polymer systems^29^. Nearest neighbour distances between CG atoms and CG atoms in other flakes are categorised as: (i) *d* $\le$ 8 Å the atom is in an “aggregated” environment, (ii) 8 Å $\le$ *d* < 18 Å the atom is an “intercalated” environment and (iii) *d* $\ge$ 18 Å the atom is in an “unbound” environment. In Fig. 2a, we show how the percentage of atoms in each environment is used to define the overall flake morphology. The four flake morphologies are designated as “intercalated”, “aggregated”, “partially aggregated” and “dispersed”.

Additionally, by comparing the number of aggregated interactions between basal plane atoms to the number of aggregated interactions between functional edge atoms, we can determine whether the assemblies contain face–face, edge–edge or face–edge interactions. Our classifications are as follows: if edge–edge aggregation is present and basal-basal interactions between the molecules are < 25% of the molecule’s basal atoms, we categorise this as edge–edge assembly. If basal–basal interactions are > 60% we classify this as face-face aggregation and if basal-basal interactions are > 25% and < 60% we classify this as partial-overlap. If aggregation interactions between basal and edge atoms are present and the basal-basal interaction is < 25%, we classify the flake as having face-edge assembly. A schematic diagram of the different binding modes is shown in Fig. 1a.

**Figure 3:**
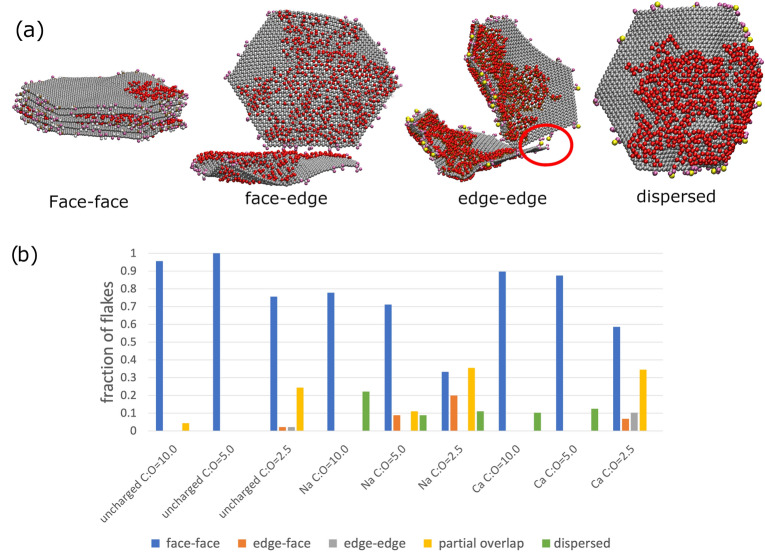
Aggregation interaction modes present in graphene oxide flakes in aqueous solution. (**a**) Snapshots from the coarse-grained simulations illustrating the relevant binding mode. The red circle on the edge-edge binding mode snapshot highlights how a $\hbox {Ca}^{2+}$ is acting as a ion bridge and facilitating the edge-edge binding mode. The colour scheme is the same as Figure 1. In (**b**), we display the percentage of flakes in each binding mode for the different systems in our study. The different binding modes (“face-face”, “partial”, “edge-edge” and “face-edge” are schematically illustrated in Fig. 1). Flakes that are not bound are classed as dispersed.

**Figure 4:**
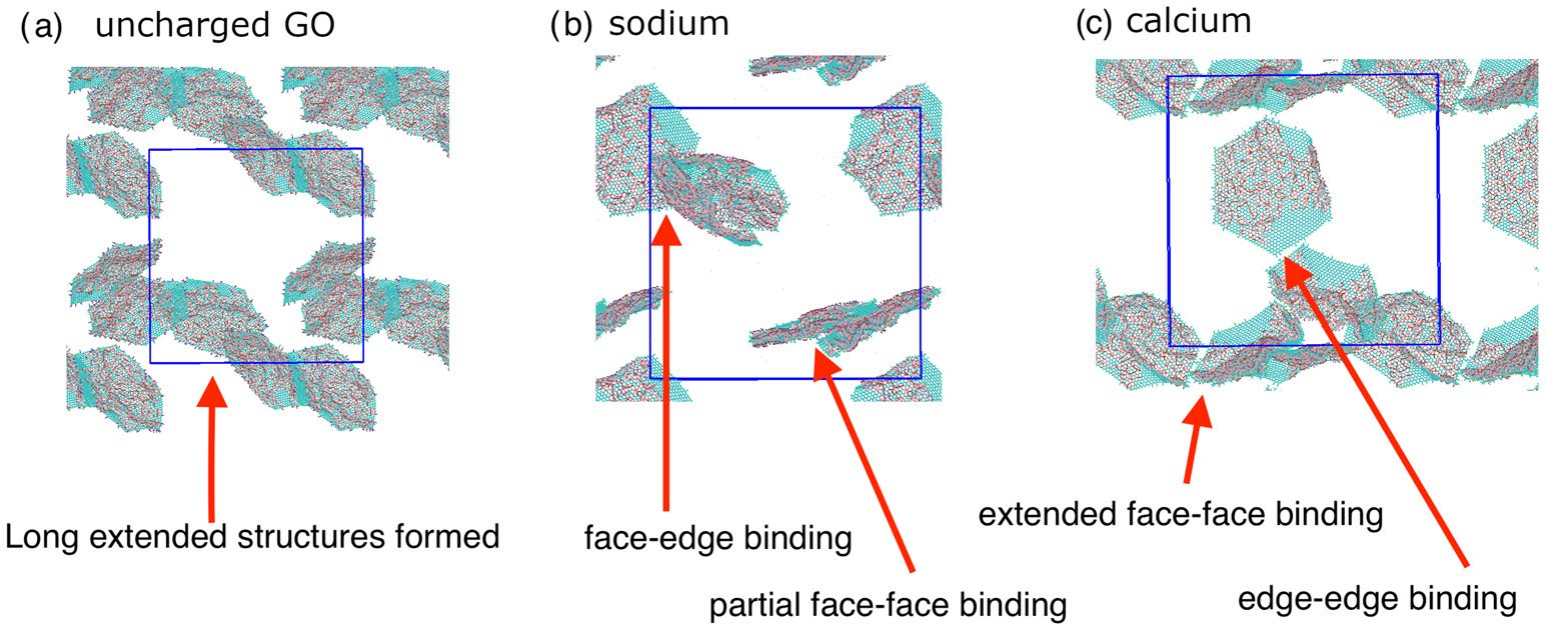
Snapshots from the atomistic simulations at 300K after the simulated annealing run, illustrating the different binding modes observed for GO with a C:O ratio of 2.5. The same binding modes are observed in the CG simulations. (**a**) For uncharged graphene oxide with protonated carboxylic acid groups, a long extended structure is formed, with aggregation over both the graphitic carbons and the oxidised regions. (**b**) For deprotonated carboxylic acid groups and sodium counterions, the GO sheets are more dispersed, but we still observe face-face binding and face–edge binding. (**c**) For deprotonated carboxylic acid groups and divalent calcium ions, we find face-face binding modes, along with edge–edge binding with $\hbox {Ca}^{2+}$ acting as an ion bridge. Water and counterions have been removed to aid visualisation. The blue lines are the periodic boundaries; we have shown atoms in the periodic replicas to better illustrate the extended structures formed.

**Figure 5:**
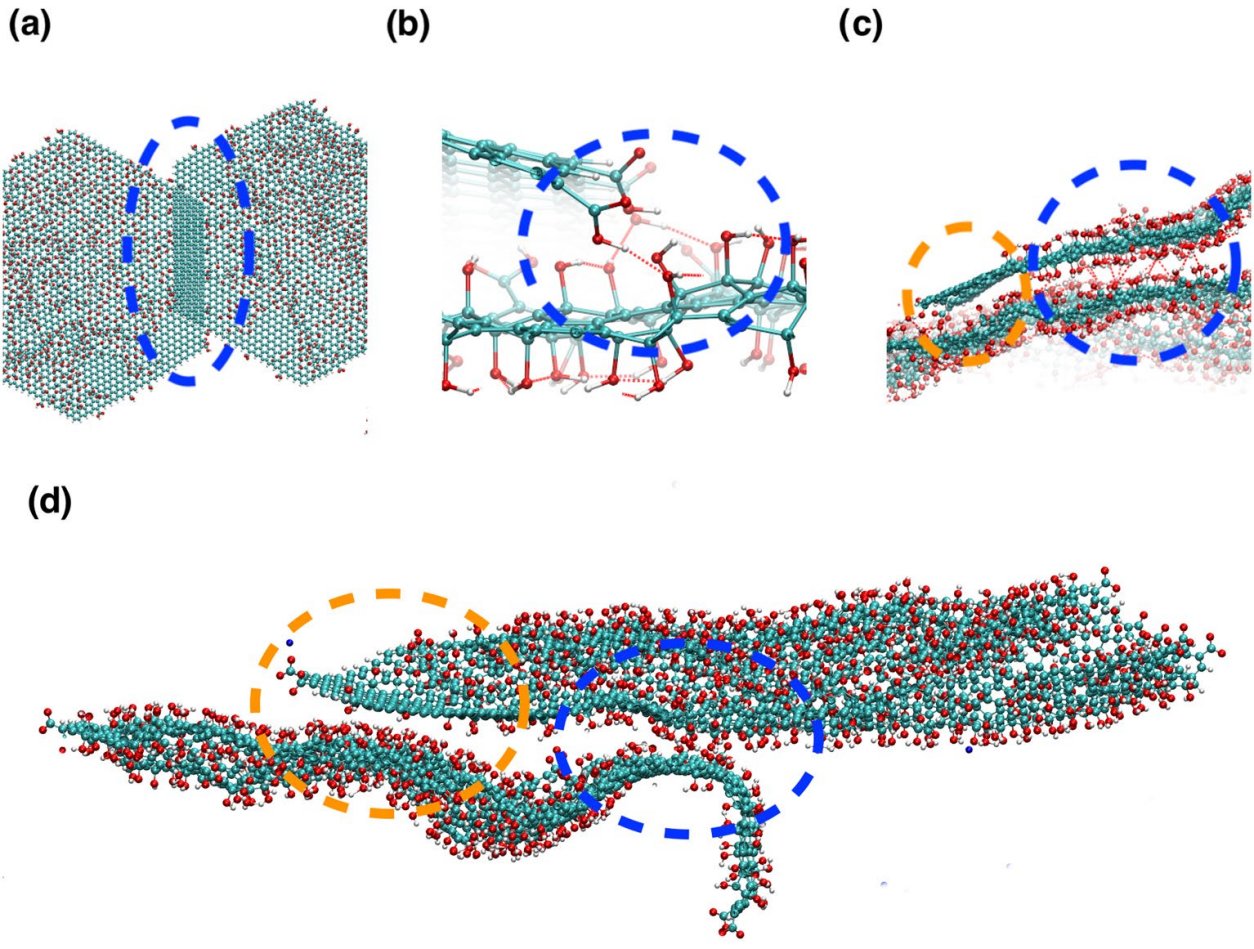
Snapshots from the atomistic simulations at 300K after the simulated annealing run, illustrating the different partial face-face binding modes observed for GO with a C:O ratio of 2.5. (**a**)–(**c**) are snapshots for uncharged (*i.e.* low pH GO), (**d**) is a snapshot for charged GO flakes with $\hbox {Na}^{+}$ ions. The different binding modes are highlighted by dashed ovals and circles. (**a**) The flakes have overlapped due to $\pi$–$\pi$ stacking interactions between $sp^{2}$ carbon atoms. (**b**) The protonated carboxylic acid atoms form hydrogen bonds with hydroxyl groups on overlapping flakes. Hydrogen bonds are shown by red dashed lines, defined by the distance between oxygen and hydrogen atoms < 4.5 Å and acceptor oxygen–donator oxygen–hydrogen atom angle < 40$^\circ$. (**c**) Blue circle: hydroxyl groups form hydrogen bonds with hydroxyl groups on overlapping flakes. Orange circle: $sp^{2}$ carbon atoms are interacting with an area of oxidised graphene oxide on an adjacent sheet, via van der Waal’s interactions. (**d**) Binding modes for two charged GO sheets with $\hbox {Na}^{+}$ ions. The orange circle highlights interactions between a $sp^{2}$ carbon atoms and hydroylated regions on an adjacent flake, the blue circle shows hydrogen bonded interactions between the flakes. The lower flake has become wavy and contorted to keep apart the charged carboxylate groups.

Starting from a dispersed initial condition, the most common flake morphology for uncharged systems, i.e. at low pH, to develop during the simulated annealing run is aggregated, irrespective of oxidation state (Fig. 2b). For oxidised systems with large graphitic regions (C:O ratios of 5.0 and 10.0), we find > 97% of flake atoms are aggregated with another flake (see Table S3). Figure 3 shows that aggregation by lowering of the pH primarily for low oxidation GO occurs in the face-face pattern because of increased van der Waals interactions between graphene carbon atoms, leading to a $\pi$-$\pi$ stacking arrangement between $sp^{2}$ carbon atoms analogous to graphite. The face-face aggregation also involves hydrogen bonds being formed between hydroxyl groups on adjacent flakes. An illustration of the graphite-like stack for C:O ratio of 10.0 is shown in Fig. 2b. This behaviour occurs due to the reduction in electrostatic repulsion at low pH, as shown by previous DLVO studies^20^.

For highly oxidised systems, such as that with a C:O ratio of 2.5, the face-face aggregation is only over part of the flake and consequently the amount of aggregated atoms is less than for C:O ratios of 5.0 and 10.0. This pattern of partial overlap is repeated with other flakes, which combine together, leading to extended 2-dimensional structures. A snapshot from a simulation illustrating this morphology is shown in Fig. 2b. None of our simulations at a C:O ratio of 2.5 formed a single, graphite-like stack, unlike for C:O ratios of 5.0 and 10.0 Visualising the morphologies formed in each of our ensembles of simulations with C:O ratio of 2.5, we always observe partially overlapped configurations, with a variable amount of overlap (see Fig. S12). In our ensembles of simulations, we find examples of flakes overlapping with a single flake, two flakes and three flakes, the last of which possesses an geometry analogous to trigonal planar geometry, with three flakes in a plane and having 120 degrees between them. Where the overlap is higher, we observe greater hydrogen bonding between the hydroxyl groups on adjacent flakes.

In addition, we observe similar binding patterns emerging during our atomistic simulations, even in the limited timescale available for atomistic simulations. Again, partial face-face binding is observed leading to an extended aggregation structure, as shown in Fig. 4a. Atomistic simulations allow us to study in detail the interactions present at the sites of partial overlap between flakes. These interactions are shown in Fig. 5, where we observe four different binding modes: $\pi$–$\pi$ stacking between the graphene $sp^{2}$ atoms on different flakes, hydrogen bonding between edge carboxylic acid groups and hydroxyl groups, hydrogen bonding between hydroxyl groups on adjacent flakes, and van der Waals interactions between the hydrophobic $sp^{2}$ carbon atoms and hydroxylated regions of overlapping flakes. We can conclude that when there are large domains of $sp^{2}$ carbon atoms, the $\pi$–$\pi$ interactions dominate, leading to full face-face aggregated structures, but when the $sp^{2}$ domains are small, the resulting other interactions are comparable to the hydrogen bonding interactions between the hydroxyl groups and water, and hence a mixture of interaction modes are seen between flakes in a partially overlapped aggregated structure. These complex interactions are revealed through our simulation, and show behaviour that can not be predicted through mean-field methods such as DLVO.

It has been observed experimentally by Chen et al. that as acid concentration increases of a solution containing GOQDs, a new PL site emerges which has been associated with self-assembled packing possessing a head-to-tail molecular arrangement (called J-type aggregation)^11^. From TEM images of the concentrated GOQDs, Chen et al. observed GOQDs of average size 3nm forming aggregates with a size of 20–30nm, and AFM images showed a height of approximately 2.5nm for the formed aggregate, suggesting that the aggregate is composed of a few single GOQDs gathered head-to-tail with partial overlapping. Similar observations of irregular growth of GOQDs at low pH has been observed by Li *et al.*^30^ and lowering the pH to 3 also causes the much larger GO to aggregate^17^. This is consistent with our simulations, which show that the partial overlapping is due to both graphitic $\pi$–$\pi$ interactions, hydrogen bonding between surface hydroxyl groups and van der Waals interactions. Our simulations also suggest that a reduction in the degree of oxidation of the GO flakes will lead to greater face-face aggregation in a stacked arrangement (H-type aggregation). This is supported by the experiments of Allahbakhsh et al.^5^, who observed a blue-shift in PL after 6 hours of the reduction process of GOQDs, which was suggested to be related to H-type aggregation to form larger particles.

At higher pH with a $\hbox {Na}^{+}$ counterion, the GO dispersion is stabilised due to the enhanced repulsion between the negatively charged edges of the GO flakes, as shown by more points at low aggregation and intercalation values in Fig. 2d. This dispersion is observed irrespective of the oxidation state of the flake. In all cases, the $\hbox {Na}^{+}$ ion moves away from the charged carboxylate ion and resides in the aqueous solution. This behaviour is expected from DLVO theory as the electrostatic repulsion is greater than the van der Waals interactions between flakes^20^. While we do find entirely separate flakes, we also observe a considerable amount of aggregation by face-face interactions, but at a reduced level compared to uncharged flakes (Fig. 3). We also observe aggregation occurring via edge-face interactions, where the carboxylic acid groups interact with the hydroxyl groups on the surface of an adjacent GO flake, as shown in Fig. 3. Our morphology classification, especially when the flakes are angled relative to each other rather than perpendicular, leads to the atoms being classified as intercalated, as they are within 18 Å of atoms on another flake. The edge-face interaction occurs predominantly in systems with a C:O ratio of 2.5, where there are a larger number of hydroxyl groups on the GO surface to interact with the carboxylate ions.

To understand why we observe face-face aggregation when the flakes are negatively charged, and therefore should be repelled from each other, we made two important observations: where face-face aggregation does occur the flakes are not fully aligned, but are sheared relative to each other, leading to the charged edge groups on adjacent sheets being > 12 Å apart. In addition, in our simulations we do not observe any more than two flakes in any stack, even for C:O ratios with large $sp^{2}$ domains. In the atomistic simulation, which has a C:O ratio of 2.5, two flakes have partially assembled (see Figs.  4 and  5d). We can identify hydrogen bonding interactions between adjacent hydroxyl groups and van der Waals interactions between the flakes. It is also clear that the flakes have become distorted from their planar geometry to avoid interactions between the charged carboxylate groups. Such bending has been reported experimentally for single layer GO^53^. This distorted geometry may hinder other flakes from aggregating to form larger particles, and therefore any further aggregation beyond two flakes is likely to be either unfavourable or a slow process. As reported by Hassanzadeh et al.^10^ and Le et al.^5^, GOQDs are a stable dispersion until their concentration is significantly increased. Our simulations suggest that the dispersion may contain aggregates of two flakes. Where TEM height functions of GOQDs are reported, for example Shin et al.^54^, heights corresponding to both mono-layer and two-layer GOQDs are found. At high concentration, we can assume aggregation occurs through the binding modes of our two-flake aggregates: i.e. hydrogen bonding and van der Waals interactions for GOQDs with high oxidation (*e.g.* C:O ratio of 2.5), and via $\pi$ – $\pi$ and van der Waals for flakes with large carbon $sp^{2}$ domains.

Replacing the $\hbox {Na}^{+}$ counterion for the divalent $\hbox {Ca}^{2+}$ ion changes the aggregation behaviour of the GO flakes, as expected from experiment^28,30^. The $\hbox {Ca}^{2+}$ ions remain bound to the nearest carboxylate ion, and effectively reduces the repulsion between sheets. With the $\hbox {Ca}^{2+}$ ions bound to their nearest negatively charged carboxylate ion, the rest of their coordination sphere is completed by water molecules, or another carboxylate ion when the $\hbox {Ca}^{2+}$ ion acts as a bridge between flakes. As described by Dimiev and co-workers, we observe the $\hbox {Ca}^{2+}$ ions form a Stern layer on the surface of the GO, rather than forming an electronically attracted diffuse layer^25^. The aggregation occurs via face-face mechanism, which is facilitated by $\hbox {Ca}^{2+}$ ions bridging between the carboxylate ions on adjacent sheets as shown in Fig. 2c. The face-face aggregation is seen for all oxidation states (Fig. 3). In a similar manner to the $\hbox {Na}^{+}$ counterion, we do not observe face-face aggregation creating stacks of greater than two or three flakes. In some of our high C:O ratio simulations, we also observe face-edge binding occurring, wherein a single flake is bound to the edge of a 2 or 3 flake stack. The $\hbox {Ca}^{2+}$ ions facilitate edge-edge aggregation, as illustrated by a simulation snapshot in Fig. 3. Here, a $\hbox {Ca}^{2+}$ ion bridges between the carboxylate groups on different flakes, while the flakes are in a partially overlapped configuration. In Fig. 4c, we show a snapshot from the atomistic simulation with a C:O ratio of 2.5 and $\hbox {Ca}^{2+}$ ions. We observe a mixture of face–face interactions leading to partial overlapping of flakes (through the same hydrogen bonding and van der Waals interactions seen for the $\hbox {Na}^{+}$ system) and edge-edge interactions via $\hbox {Ca}^{2+}$ cation bridging. While the face-face interactions require the flakes to remain approximately planar, the edge-edge interactions do not, with the end result a more open 3D structure with planar face-face aggregates joined together by $\hbox {Ca}^{2+}$ cation bridging.

Experimentally, addition of calcium ions to a GOQDs dispersion significantly increases aggregation^30^, as shown by SEM images, where irregular aggregated shapes were observed. The combination of $\hbox {Ca}^{2+}$ increasing aggregation through both face–face and edge–edge binding is believed to be the reason^55^ for the enhancement of mechanical properties of GO sheets with a small amount of $\hbox {Mg}^{2+}$ and $\hbox {Ca}^{2+}$.

## Conclusions

Understanding the relationship between flake composition and structure is critical for optimising the performance of aqueous graphene oxide and graphene oxide quantum dot systems. Our simulations, which contain realistic domains of oxidized and non-oxidized regions, have shown that these domains are very important for the aggregation of GO and GOQDs. We have observed a variety of different interactions between aggregated sheets, with regions with high density of oxidation forming hydrogen bonds with neighboring flakes, while $\hbox {sp}^{2}$ carbon regions form graphite-like $\pi$-$\pi$ stacking.

From our findings, we can specify a set of general rules relating the composition of the flakes to the morphologies formed and binding modes present when under thermodynamic control, as follows:

*Rule 1*: Low pH causes the carboxylic acid edge groups to be protonated and the flakes will aggregate in a face-face binding mode, as seen in experiment and confirming the reduction of electrostatic repulsion shown in DLVO studies.

*Rule 2*: At low pH the structure of the assembled flake depends upon the degree of oxidation on the surface; highly oxidised GO form planar aggregates of overlapping flakes that bind through a variety of secondary interactions. These include hydrogen bonds forming between hydroxyl groups on different sheets, overlap of $\hbox {sp}^{2}$ regions and interactions between carboxyl acid groups and hydroxyl groups on different flakes. To emerge from our simulations, these interactions must, therefore, be comparable, or stronger, than interactions of these groups with water alone.

*Rule 3*: High pH with sodium counterions supports the formations of stable dispersions of one or two flakes, as seen in experiment and predicted from DLVO studies.

*Rule 4*: Hydrogen bonding between areas of high hydroxyl density and van der Waals interactions between $sp^{2}$ and oxidised domains are strong enough to cause some highly oxidised GO flakes of the same charge to assemble into tactoids containing 2 flakes, despite the electrostatic repulsion of the flakes. This unexpected result illustrates the large number of strong bonds being formed between oxidised regions. The assembled flakes only partially overlap, forming curved configurations due to electrostatic repulsion of carboxylate groups.

*Rule 5*: Calcium ions at high pH cause the graphene oxide sheets to aggregate in a face-face binding mode with some edge-edge binding, both promoted via cation bridging, creating irregular 3D structures. The $\hbox {Ca}^{2+}$ ions are bound to a carboxylate ion with the rest of the coordination sphere being either water molecules or other carboxylate ions.

Our multiscale simulations furnish a major advance in our understanding of GO and GOQD aggregation and dispersion in aqueous solution. Building on our previous study of graphene oxide in polymeric media, future work will seek principles by means of which we can understand how other surface functionalisations found on the GO surface, including epoxies, peroxide groups, sulfates and defects, affect the properties of GO^56^. In addition, we will investigate combining GO with aqueous polymers to further control the formation of self-assembled GO to create targeted structures on the nano- and meso-scale for nanocomposite applications.

## Supplementary Information

Supplementary Information.

## Data Availability

The Supporting Information is available from the Scientific Reports website or directly from the authors. The data that support the findings of this study are openly available in Zenodo at 10.5281/zenodo.4919597.
