# Supplementary material for: Principles governing control of aggregation and dispersion of aqueous graphene oxide
Source: Sci Rep. 2021 Nov 17;11:22460. doi: 10.1038/s41598-021-01626-3 (PMC8599484; doi:10.1038/s41598-021-01626-3)
Supplement: Supplementary file 1 — Supplementary Information. [file 41598_2021_1626_MOESM1_ESM.pdf]

Supporting Information: Principles Governing Control of Aggregation and Dispersion of Aqueous Graphene Oxide

James L. Suter 0000-0002-0149-7974[†] and Peter V. Coveney 0000-0002-8787-7256[*,†,‡]

†*Centre for Computational Science - University College London, 20 Gordon Street, London, WC1H 0AJ, United Kingdom*

‡*Computational Science Laboratory, Institute for Informatics, Faculty of Science, University of Amsterdam, 1098XH, The Netherlands*

E-mail: p.v.coveney@ucl.ac.uk

In this Supporting Information (SI), we report in detail the methodology we have employed in constructing our multiscale aqueous graphene-oxide simulation systems.

To represent water molecules, we have used the Stillinger-Weber (SW) potential[1] (which is called mW water)[2]. This potential represents water molecules as single interaction sites, which interact via two- and three- body potentials. The SW potential has been shown to accurately model water behaviour, as well as aqueous-salt interactions[3] and water-soluble polymers[4]. Compared to full atomistic water, such as TIP4P, the reduction in the number of interaction points, along with the short-range potentials and no electrostatics, leads to speed-up of around 65x[2].

In our study, we have utilised the SW models developed by Islam *et al.* for protonated

and deprotonated carboxylic acids, which were subsequently applied to poly(methacrylic acid)[5]. For the non-bonded interactions that are not previously described by the parameter set of Islam *et al.*, we have used a combination of techniques to construct the CG effective pair potentials (which we refer to as interaction potentials), all of which are constructed to reproduce selected properties of fine-grained (atomistic) simulations. These include matching to potentials of mean force and radial distribution functions.

To construct our model of GO, we have used a hybrid atomistic coarse-grained approach. In Section S1, we describe the mapping implemented between the atomistic representation and the hybrid coarse-grained system. In Section S2, we list the simulations we have performed, both at the atomistic (all atom, AA) and CG level, to create all the parameters required to describe these complex systems. Finally, in Section S3, we list our procedure for generating the aqueous graphene oxide systems.

S1 Atomistic to coarse-grained particle mapping

In this section we describe the mapping operator that transforms selected groups of atoms into CG particles.

sp^2 carbon atoms of the GO framework have a 1:1 mapping to CG atoms, and interact with other sp^2 carbon atoms using the OPLS potential, in the same manner as atomistic graphene. As these atoms do not possess any partial charge in the OPLS forcefield, non-bonded interactions between sp^2 carbon atoms act only via Lennard-Jones interactions. Through representing the sp^2 carbon atoms in their atomistic form, we preserve the flat nature of the graphene flakes; there is no possibility of a corrugated potential energy surface above and below the graphene surface, which could result when multiple carbon atoms (e.g. 4) are mapped to a single CG bead. This CG bead type is referred to as type **C** from here on.

We have used a united atom approach to generate the edges of the graphene flakes, by

mapping the edge carbon and hydrogen atoms into a single CG bead. We have used the OPLS forcefield parameters for sp^2 carbons atoms to represent the graphene edge CH unit (referred to as type **CH** from here on). Each hydroxyl group on the graphene surface is represented by a single united atom that maps to the the oxygen and the hydrogen atoms (type **OH**). The carbon atom bound to the oxygen is referred to as type **COH**. For the carboxylic acid groups on the edge of the graphene sheet, we have used the naming convention of Islam *et al.*[5]. These atoms types are **CO**, **COA**, **OH**, **O**, and **OA**, where **OH** refers to the protonated oxygen, **O** to the oxygen with the double bond, **OA** to the carboxylate oxygen, **CO** to the carbon of the protonated acid, and **COA** to the carbon atom of the deprotonated acid. There is a 1:1 mapping of AA to CG atoms for the counterions Na^+ and Ca^+.

The mapping between atomistic and coarse-grained representations is shown in Figure S1.

S2 Derivation of coarse-grained force field parameters

To create a full set of interaction potentials between all the coarse-grained atom types described in the previous section we have used a variety of techniques. In Figure S2, we show the correspondence between the interaction type and the technique used to generation the interaction potential. Using this workflow, we build upon each successive generation of potentials, until all potentials are defined for the aqueous GO systems described in the main paper. The SW parameter set of Islam *et al.* provides the interactions between all mW water and carboxylic acid types. The full list of SW parameters is listed in reference[5]. More details on how we implemented the SW parameter set can be found in section S2.1. The carbon atoms of the graphene sheet (i.e. **C**, **COH** and **CH**) have zero or very low charge in the OPLS forcefield, and their interaction potentials are approximated by the Lennard-Jones potential, using parameters from the OPLS forcefield. Their interactions with **OA**, O, **OH**, **COA**, **Ca** and **Na** are computed using the Lorentz-Berthelot mixing rules between the respective OPLS forcefield parameters. The cutoff for these potentials is 12 Å. The

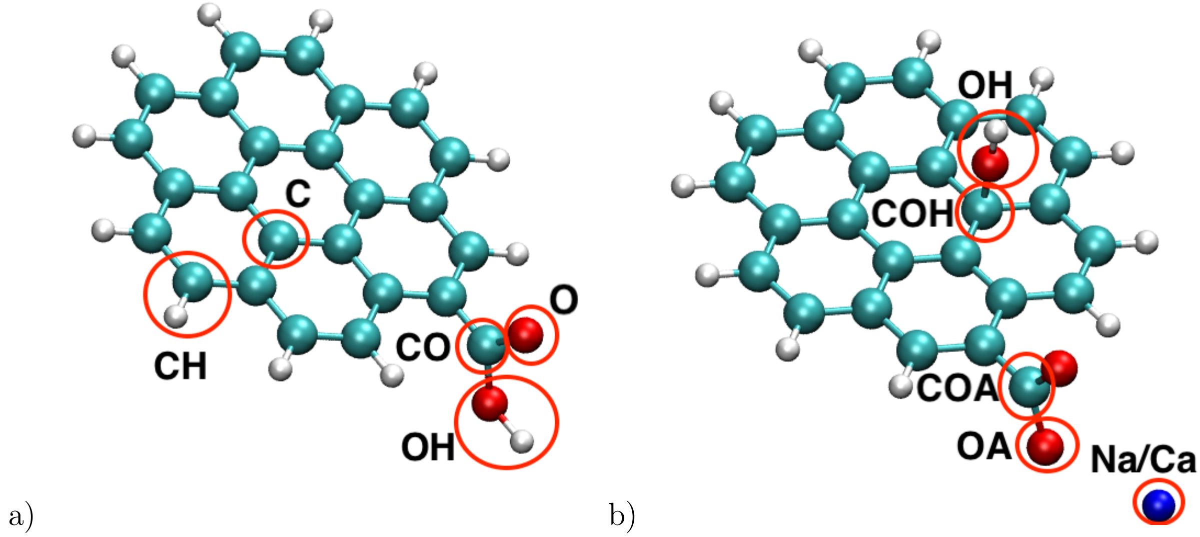

Figure S1: Schematic illustration of coarse-grained (CG) graphene oxide (GO) mapping. The Figure shows the all-atom (AA) GO structure and the AA to CG mapping scheme. The colouring scheme is as follows: carbon atoms are green, oxygen atoms are red, hydrogen atoms are white and sodium / calcium ions are blue. The red circles indicate the atoms that each CG bead maps to. In a), we show the mapping of four types of CG beads (non-oxidized sp^2 carbon (**C**), edge non-oxidised carbon (**CH**), the carboxylic acid protonated oxygen (**OH**), and the double bonded carbonyl oxygen (**O**)). In b), we show the mapping for the following CG types: hydroxyl groups on the graphene surface (**OH**), carbon atoms bound to hydroxyl groups (**COH**), the carboxylate oxygen (**OA**), the carbon of the deprotonated carboxylic acid (**COA**) and the counterion (either $\mathbf{Na}^+$ or $\mathbf{Ca}^{2+}$).

Lennard-Jones parameters can be found in Table S1.

To create the CG interaction potentials between mW water and graphene carbon atoms, we used a small atomistic simulation of graphene in water and matched the density profiles perpendicular to the graphene surface. This technique for generating interaction potentials is described in Suter *et al.*[6] and the results are shown in section S2.2.

Furthermore, to create the interactions between ions (i.e **OA-OA**, **Ca-OA** etc) we have created interaction potentials by matching to potentials of mean force (PMF) between the ions. The results of matching to PMFs are described in section S2.3.2.

All non-bonded interactions (bonds, angles, dihedrals and impropers) for the GO flake

at the CG level are described using the atomistic OPLS forcefield where there is a 1:1 AA to CG mapping, and for other CG atoms type that map to more than one atom (**OH**, **CH**), the non-bonded interactions are described by the OPLS forcefield for the heavy atom in the mapping (*i.e.* O and C respectively).

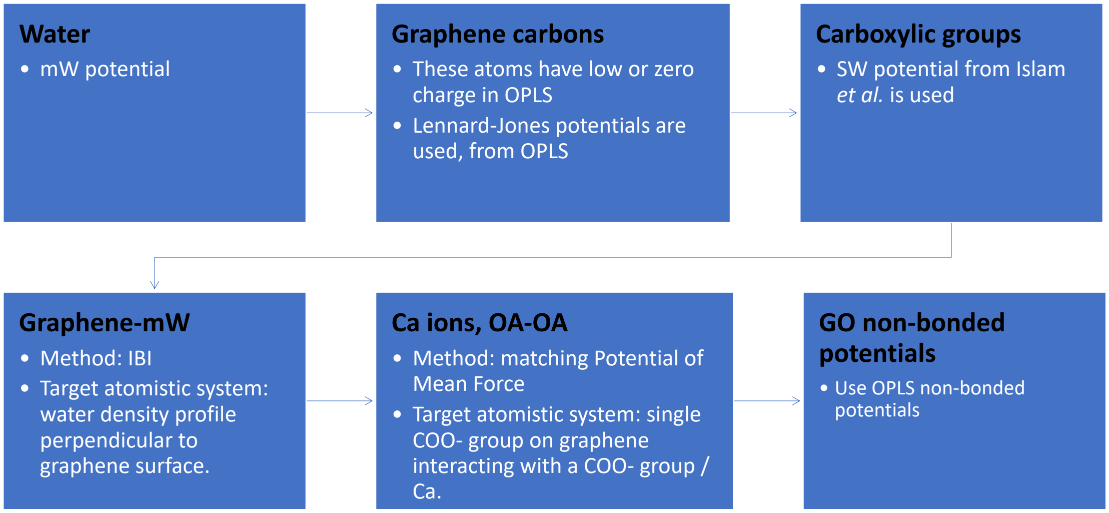

Figure S2: A schematic diagram illustrating the workflow used to compute the large number of interaction potentials required to simulate the coarse-grained GO-water systems described in the main paper.

S2.1 Stillinger-Weber potentials

All Stillinger-Weber (SW) interactions used in this study are taken from the parameter set of Islam *et al.*[5].

The energy for the SW potential is a combination of 2- and 3- body terms (ϕ_2 and ϕ_3)

Table S1: The Lennard-Jones interaction parameters for graphene CG atoms **C**, **CH** and **COH**). The table includes the parameters for the other CG atoms types which are combined using the Lorentz-Berthelot combination rules to create the interactions for the graphene CG atoms.

atom type	epsilon (kcal/mol)	sigma (Å)
**C**	0.07	3.55
**CH**	0.07	3.55
**COH**	0.066	3.5
combined with:		
**OA**	0.21	2.96
**O**	0.21	2.96
**OH**	0.17	3.12
**COA**	0.105	3.75
**CO**	0.105	3.75
**Ca**	0.4496	2.412
**Na**	0.0005	4.07

as follows

$$
\begin{aligned}
E &= \sum_i \sum_{j>i} \phi_2(r_{ij}) + \sum_i \sum_{j\neq i} \sum_{k>j} \phi_3(r_{ij}, r_{ik}, \theta_{ijk}) \\
\phi_2(r_{ij}) &= A_{ij}\epsilon_{ij}\left[B_{ij}(\frac{\sigma_{ij}}{r_{ij}})^{p_{ij}} - (\frac{\sigma_{ij}}{r_{ij}})^{q_{ij}}\right]\exp\left(\frac{\sigma_{ij}}{r_{ij}-a_{ij}\sigma_{ij}}\right) \\
\phi_3(r_{ij}, r_{ik}, \theta_{ijk}) &= \lambda_{ijk}\epsilon_{ijk}\left[\cos\theta_{ijk} - \cos\theta_{0ijk}\right]^2 \exp\left(\frac{\gamma_{ij}\sigma_{ij}}{r_{ij}-a_{ij}\sigma_{ij}}\right)\exp\left(\frac{\gamma_{ik}\sigma_{ik}}{r_{ik}-a_{ik}\sigma_{ik}}\right)
\end{aligned} \tag{1}
$$

The summations in the formula are over all neighbours j and k of atom i within a cutoff distance a; r_{ij} is the distance between atoms i and j and θ is the angle between atom i and its neighbors j and k.

The parameters A, B, a_{ij}, γ are taken from Islam *et al.* and are the same for all SW interactions (A=7.049556277,B=0.6022245583,a_{ij} =1.80). The well depth (ϵ) and length scale (σ) parameters for ϕ_2, the angle (θ_0) and energy scale (ϵ_{ijk} and λ_{ijk}) parameters for ϕ_3 are specified in the paper by Islam *et al.* for interactions between CG atom types **CO**, **COA**, **OH**, **O**, **OA**, **Na** and mW water.

The repulsive interaction between Na^+ ions is described in the parameter set of Islam *et*

al. via a Yukawa potential,

$$E = A\frac{e^{-\kappa r}}{r} \qquad r < r_c \tag{2}$$

with γ= 1.20, κ = 1.80 Å^{-1}, and r_c = 7.0 Å and A = 1107 kcal mol^{-1}.

To test the suitability of these parameters for use with GO flakes with carboxylic acid groups, an atomistic simulation of a single graphene oxide flake with a C:O ratio of 2.5, immersed in water, was run using OPLS and TIP4P/2005 water. This was subsequently coarse-grained and the radial distribution functions between the GO flake atom types and water were compared. An illustration of the initial setup of the test system is shown in Figure S3. The graphene-oxide flake contained 1126 atoms and is surrounded by 8198 water molecules (25720 atoms in total). One in four edge groups were randomly selected and converted to neutral carboxylic acid groups. This system was simulated at 300K and 1 atmosphere pressure for 8.5ns. The average L_x, L_y and L_z lattice dimensions were 61 Å. The coarse-grained system contained 9052 atoms and was run for 4ns. To calculate the radial distribution functions for the deprotonated carboxylate atom types (**COA** and **OA**), the same simulation set up was used with deprotonated carboxylate ions as the edge groups and Na^+ added as a counterion.

The radial distribution functions between the GO flake atom types and water molecules is shown in Figure S4. The CG model combining the parameter set of Islam *et al.*, the Lennard-Jones parameters for the zero or low charge graphene atoms and the water-graphene carbon potential created by using the Iterative Boltzmann Inversion method (see below) with mW water is in reasonable agreement with the atomistic simulations, capturing the position of the first hydration shells and their peak heights. However, we altered the interaction between the **OH** atom on the carboxylic acid group and water to better match the first peak in the g(r); the updated parameters are ϵ = 0.740 and σ = 2.497.

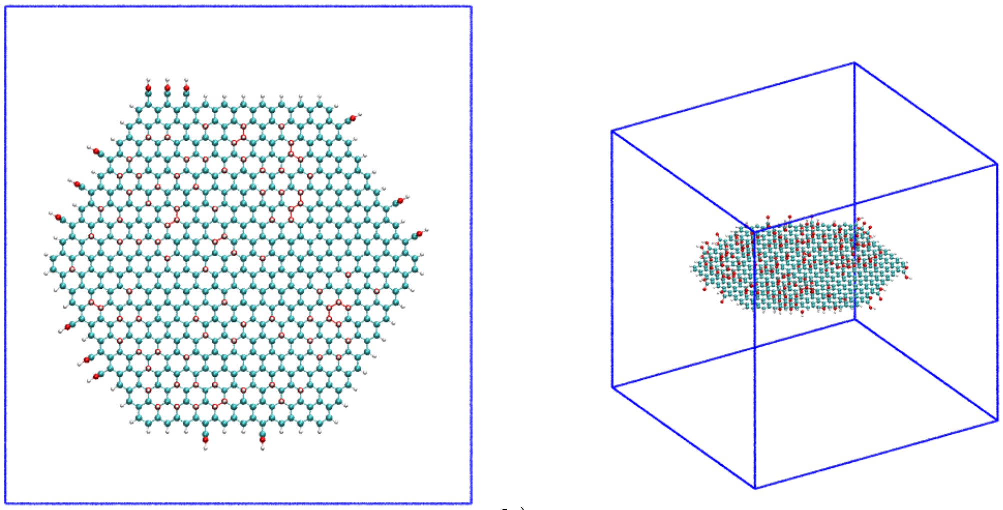

a) b)

Figure S3: Illustration of the test simulation setup for a single GO flake in water. The blue lines represent the periodic boundaries of the simulation cell, which is cubic with L_x=L_y=L_z= 61 . The radius of the GO flake is approximately 50 Å. Water molecules have been removed to aid visualisation. The colour scheme is as follows: oxygen atoms = red, hydrogen atoms = white, carbon atoms = green.

S2.2 Graphene-mW surface interactions

At the coarse-grained level, the interaction energy between the GO sheet and water molecules are represented by an interaction potential; as we require flexible (and indeed mobile) GO sheets we cannot use a simple z-dependent potential (with the graphene oxide sheets as walls fixed on the xy plane).

The adsorbed water is best described by the density profile perpendicular to the surface. This captures the adsorption properties such as the adsorbed layer thickness and density. We therefore derive coarse-grained water-graphene interaction parameters which reproduce the atomistic density profile perpendicular to the surface. Our target atomistic system is a graphene sheet lying in the xy plane, with periodic boundaries located on the graphene sheet, and water molecules above and below the sheet.

To compute the interaction potential between mW water and graphene **C** CG atoms, we simulated a reference atomistic model of a periodic graphene with water above and below the graphene sheet. A snapshot from this simulation is shown in Figure S5a. Coarse-grained

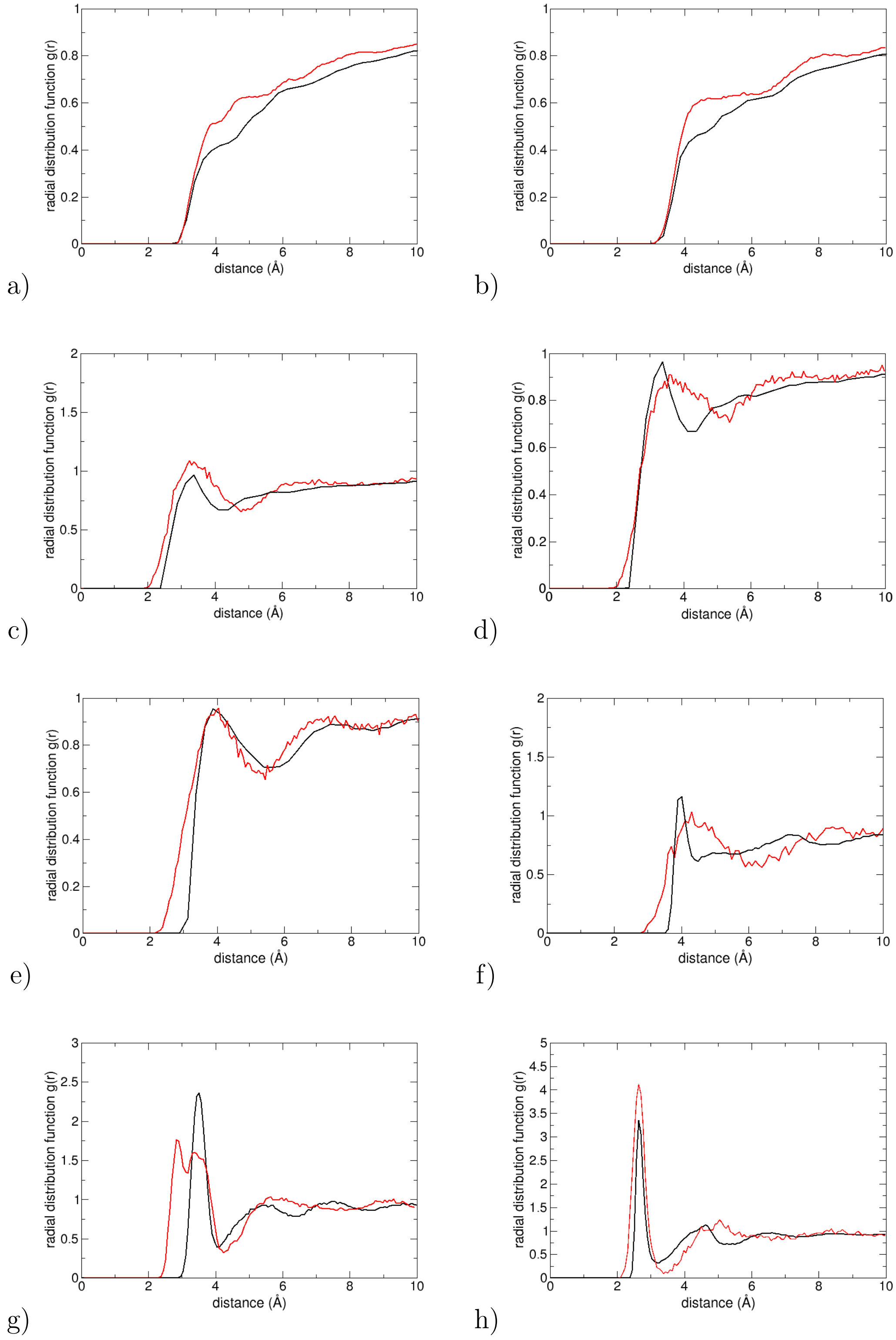

Figure S4: Comparison of the radial distributions between water and atom types on the GO flakes for atomistic (black) and coarse-grained equivalent (red) representations. The RDFs are between water and the following atom types: a) **C**, b) **COH**, c) **O**, d) hydroxyl **OH**, e) **CO**, f) carboxylic acid **OH**, g) **COA** and **OA**.

potentials were computed as described in our previous publication [6]. The matching density profiles for water perpendicular to the graphene surface are shown in Figure S5b. The resulting tabular potential was used for the following interactions: mW water with **C**, **COH** and **CH**.

Atomistic details: 10146 atoms. The system contained 3203 water molecules and was simulated in the NpT ensemble (1 atmosphere and 300K). The average lattice parameters were $36.4 \times 37.8 \times 73.5$ Å^3. The temperature was maintained at 300K and 1 atmosphere, and was simulated for 17,200,000 timesteps at 1.5fs (25.8ns), with bonds between water oxygen and hydrogen atoms constrained to their equilibrium values using SHAKE. The coarse-grained system was run in a NVT ensemble, with lattice parameters corresponding to the average lattice parameters for the atomistic system. The coarse-grained system contained 3742 CG atoms. Each coarse-grained iteration was run for 12.5 ns with a timestep of 5fs.

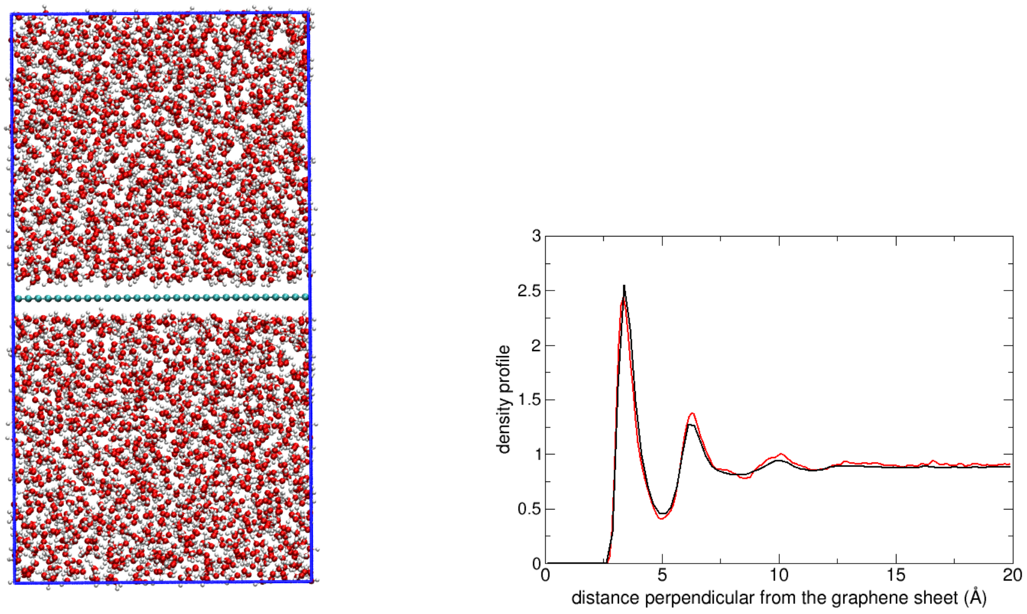

Figure S5: The atomistic reference system and its coarse-grained equivalent for parameterising the mW water-**C** interaction potentials. The left hand panel is an atomistic simulation of a graphene sheet with water molecules in the xz plane. The colours are as follows: carbon atoms are green, oxygen red and hydrogen white.

S2.3 Ca ions, OA-OA interactions

S2.3.1 Ca-water interactions

To calculate the coarse grained interaction between calcium ions and water, we used the Iterative Boltmann Inversion (IBI) method to match the atomistic radial distribution between the Ca^{2+} ion and water molecules. The IBI method has been descibed in detail in our previous publication[6]. The matching distributions, within an error of 0.05, are shown in Figure S6. **Atomistic details:** A single Ca^{2+} ion was inserted in a box of water of dimensions 29.2 Å ^{3}. The Ca^{2+} ion was described using the OPLS forcefield, with TIP4P for the water molecules. The simulation box contained 891 water molecules and was simulated for 3ns using a NPT ensemble at 300K. The calcium ion formed a first hydration shell with 8 water molecules at a distance of 2.48 Å. This tight binding of water molecules to calcium is well known with the OPLS forcefield. For the repulsive interaction between Ca^{2+} ions, we have used the (repulsive part of the) Buckingham potential as described by King *et al.* in their coarse-grained simulation of $CaCO_3$ aggregation and crystallization with mW water (A = 1295349217 kcal mol^{-1}, σ = 0.1760986 Å, C = 0.0 kcal mol^{-1} and r_c = 10 Å[7]). **Coarse-Grained details:** 892 atoms, run for 10ns with mW water.

S2.3.2 OA-OA and Ca-COA interactions

To calculate the **OA - OA** and **Ca - COA** interaction potentials, we matched the atomistic and coarse-grained potential of mean force (PMF) for a graphene flake containing a single COO^- group interacting with another flake containing a single COO^- group or a Ca^{2+} ion respectively.

An illustration of this setup is shown in Figure S2.3.2, where the arrow indicates the reaction coordinate. The PMF calculation is achieved using umbrella sampling, which requires an ensemble of simulations, each constrained to a value on the reaction coordinate using a harmonic potential. The PMF can then be reconstructed using the weighted histogram anal-

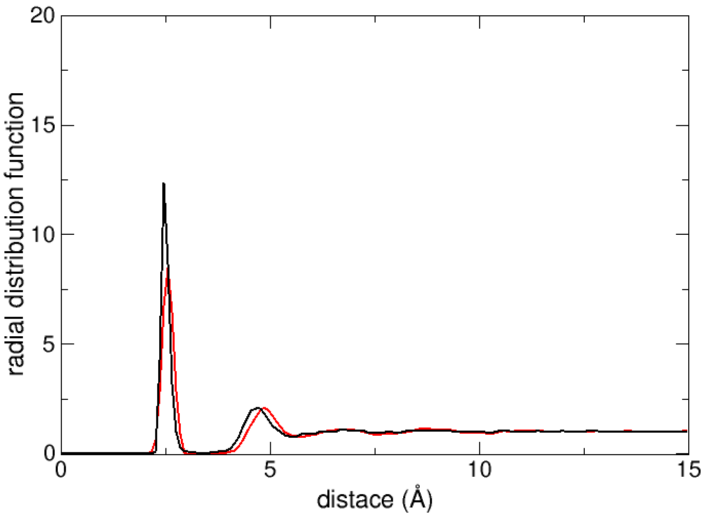

Figure S6: Comparison of the radial distribution function between Ca and water at the atomistic level (black) and the CG level (red) after optimisation of the **Ca**-mW water potential using the IBI method.

ysis method (WHAM)[8]. The tabular coarse-grained interaction potentials generated through matching to the atomistic PMF have a spacing of 0.15 Å and a cutoff of 15Å, reflecting the long-range nature of interactions between charged species.

S2.3.3 OA-OA interactions

Atomistic details: The atomistic reference system contains two graphene flakes comprised of 24 graphene C atoms and one COO^- group, as shown in Figure S7. The graphene flakes were immersed in a box of TIP4P water molecules. The box size is approximately 50 Å^3, containing 12453 atoms. Two additional charge balancing Na^+ ions were added to the system at a distance > 25 Å from the graphene sheet. The final snapshots were checked to ensure the Na^+ ions were still over 20 Å away from the COO^- groups. The distance between the **COA** atoms is used as the reaction coordinate. The reaction coordinate is varied between 15 Å and 2.55 Å (83 replicas at different reaction coordinate values, varying in steps of 0.15Å). The constraint value was enforced using an harmonic constraint of 4.7 kcal Å^{-1}. Each replica

was run for 0.1ns at 300K and 1 atmosphere pressure. The final snapshots were checked that the sheets remained relatively colinear and had not assembled over the graphene surface. **Coarse-Grained details:** 4179 atoms. Each replica was run for 0.2ns.

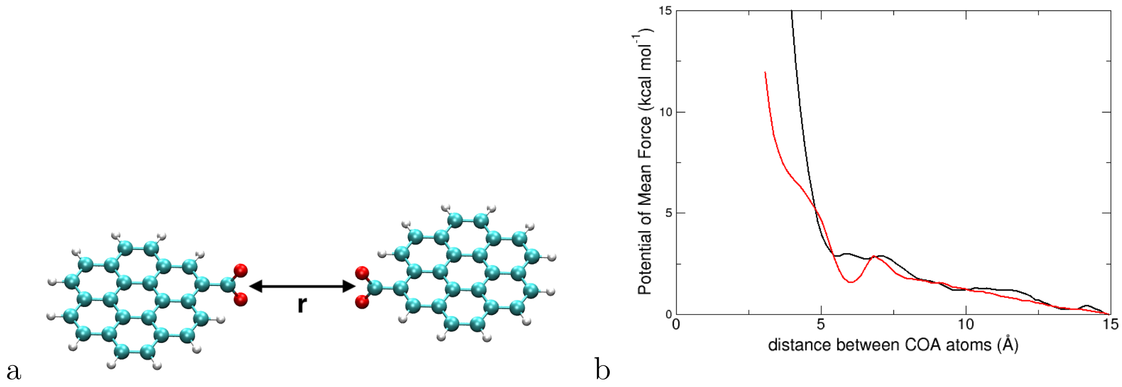

Figure S7: Illustrations of the potential of mean force calculations used to compute the a) **OA** - **OA** interaction potential and b) the corresponding **OA** - **OA** PMF at the atomistic level (black) and CG level (red). The black arrow in a) is the reaction coordinate for the PMF.

S2.3.4 Ca-OA interactions

Atomistic details: The reference system contains a single graphene flake composed of 24 graphene C atoms with a COO^- group and a Ca^{2+} ion. The reaction coordinate for the PMF is the distance between the Ca^{2+} ion and the COA atom, as shown in Figure S8a. To simplify the interaction between the COO^- group and the Ca^{2+} ion, at the CG level the interaction is for the **Ca** is only via the **COA** atoms, while interactions between **OA** and **Ca** are neglected. The interactions of the OA atoms with the Ca ion are therefore included in the **COA-Ca** effective potential. The atomistic system contains 6375 atoms (2116 water molecules) and 83 replicas were simulated, with constraint values varying between 15 Å and 2.55 Å. Each replica was run for 2ns at 300K and 1 atmosphere pressure. The average L_x, L_y and L_z lattice dimensions were 40.2 Å. Each coarse-grained replica contained 2140 atoms and was run for 2ns.

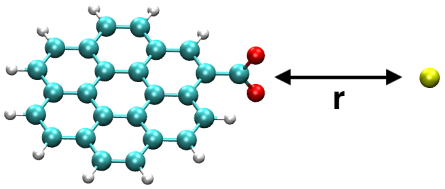

a

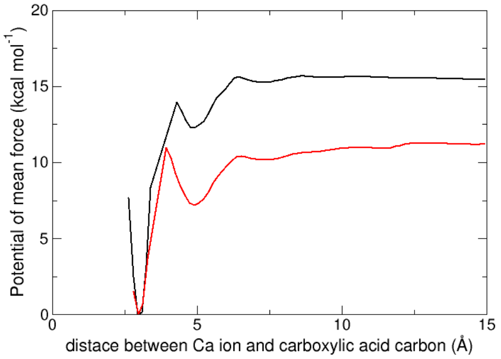

b

Figure S8: Illustrations of the potential of mean force calculations used to compute the a) **Ca** - **COA** interaction potential and b) the corresponding **Ca** - **COA** PMF at the atomistic level (black) and CG level (red). The black arrow in a) is the reaction coordinate for the PMF.

S2.3.5 Test system

To test whether the combination of **Ca** CG potentials produces the same behaviour at both atomistic and coarse-grained level, we performed a potential of mean force calculation between two graphene sheets with COO^- groups (analogous to the PMF simulation to calculate the **OA-OA** interaction potential) with a Ca^{2+} ion acting as an ion bridge between the two COO^- groups. An illustration of this setup is shown in S9a. This system contained 12452 atoms (4125 water molecules), with 75 replicas at different constraint values with a 0.15 interval, varying from 15.0 Å to 3.75 Å. The average L_x, L_y and L_z lattice dimensions were 50.2 Å and each simulation lasted 4ns. This long simulation time allowed the graphene sheets to assemble over the graphitic regions, both in the atomistic and coarse-grained simulations. As can be seen from Figure S9b, the PMFs generated are similar, with a larger barrier to flake separation. The atomistic simulation has a shallower potential well, presumably due to the coarse-grained **Ca** ion only interacting with the COO^- group via the **COA** atom, which allows more less energy configurations to be attained at low constraint values. However, this test PMF simulation shows that the energy difference of two charged flakes interacting with Ca^{2+} acting as a cation bridge is broadly the same in both atomistic and coarse-grained

representations.

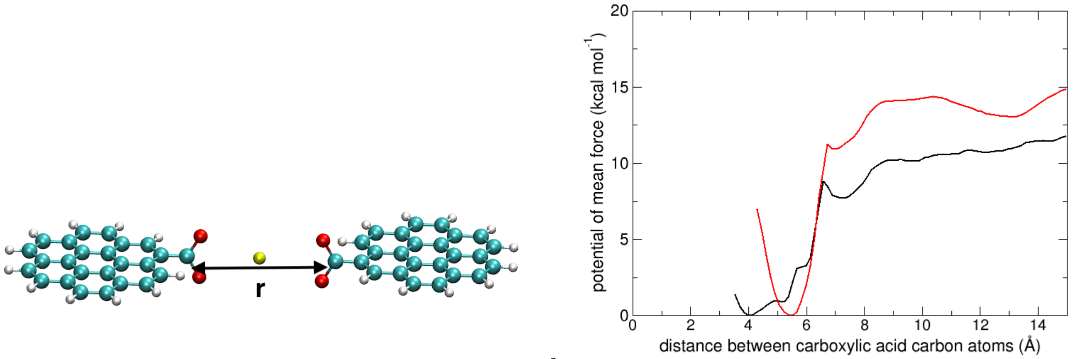

a b

Figure S9: a) Illustration of the potential of the test potential of mean force simulation used to examine the behaviour of the Ca^{2+} ion as a cation bridge. The black arrow is the PMF reaction coordinate, which corresponds to the distance between the COA atoms. The Ca^{2+} ion is initially between the two COO^- groups. b) The resulting PMF at the atomistic (black) and CG level (red).

S2.3.6 Ca-OH interactions

To calculate the effective interaction between **Ca** and **OH** (hydroxyl groups on the basal surface of the GO flake), we computed a PMF between a surface OH group and a Ca^{2+} ion, which was started directly above the OH group on the GO flake. An illustration of this set-up is shown in Figure S10a. The corresponding PMF is shown in Figure S10b, which shows that, due to large binding energy between water and Ca^{2+}, the effective potential is repulsive, as the Ca^{2+} would rather be in solution that bound to a hydroxyl on the surface.

Atomistic details:: the atomistic system contains 6375 atoms (2112 water molecules) and 83 replicas were simulated, with constraint values varying between 15 Å and 2.55 Å. Each replica was run for 0.2ns at 300K and 1 atmosphere pressure. The average lattice dimensions were 39.7 Å. Each coarse-grained replica contained 2138 atoms and was run for 5ns.

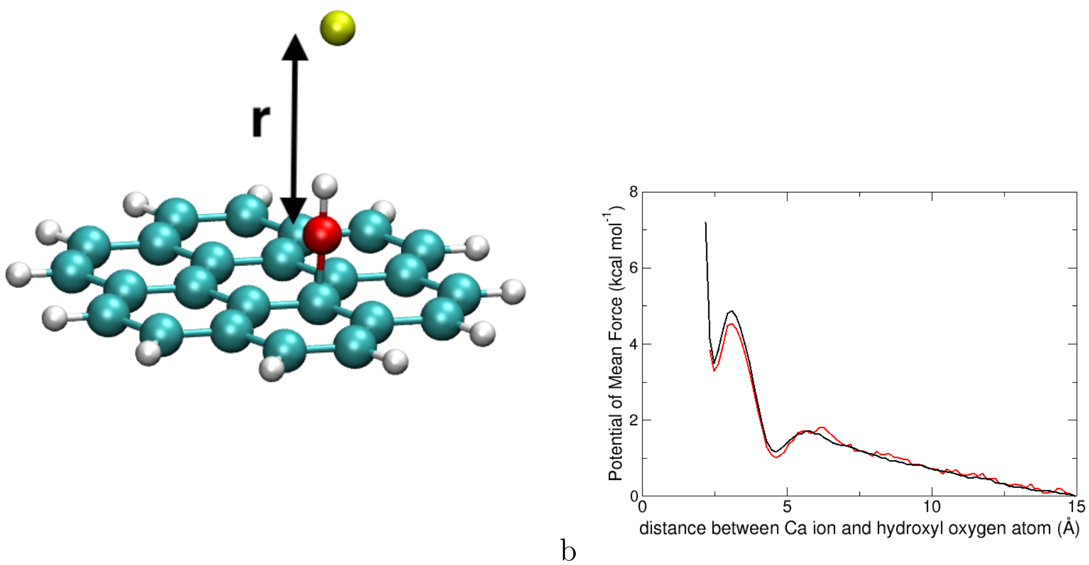

a b

Figure S10: a) illustration of the potential of mean force calculations used to compute the **Ca - OH** interaction potential The black arrow is the reaction coordinate for the PMF. b) The resulting PMF at the atomistic (black) and CG level (red).

S3 Protocols for the CG-MD production simulations

The procedure for creating the coarse-grained models described in the main paper is illustrated in Figure S11. Each model is composed of 5 flakes. Each graphene (oxide) flake is of hexagonal shape with a diameter of approximately 100 Å; in total they comprise approximately 2 - 3% of the volume of the simulation cell. We generate the oxidised structures using the procedure described in our previous publication[9–11]. To summarise this procedure, the algorithm generates graphene oxide nanostructures for atomistic simulation based on theoretical and experimental evidence[10], which improves upon the current standard approach of an uncorrelated random distribution of oxidised functional groups, based on the Lerf-Klinowski model[12]. Our algorithm uses the quantum mechanical (QM) simulations of Yang *et al.*[13], which studied the reactivity of various reactive intermediate structures and showed that graphene oxidation is overwhelmingly favoured adjacent to already oxidised carbons. As can be seen in Figure 1 of the main paper, our algorithm creates atomistic structures of GO that successfully recreate the two-phase nature of oxidised and unoxidised graphene

domains observed in microscopy experiments, and is necessary to reproduce GO assembly due to the self-attraction of graphene domains.

Here we are only considering oxidation on the basal surface consisting only of hydroxyl groups. To oxidise the edge groups, we selected 1/4 of the edge carbon edge atoms and these were converted to (protonated) carboxylic acid groups, or carboxylate ions with either a Na^+ or a Ca^{2+} ion placed 5 Å away, depending on the GO flake structure reauired. Having generated our atomistic representation of a single graphene (oxide) sheet at the required carbon / oxygen ratio and with the required carboxylic edge groups, we add five flakes to an empty simulation cell of 200 × 200 × 200 Å^3. To create initially dispersed flakes, these sheets are randomly added to the box, ensuring no overlap between atoms.

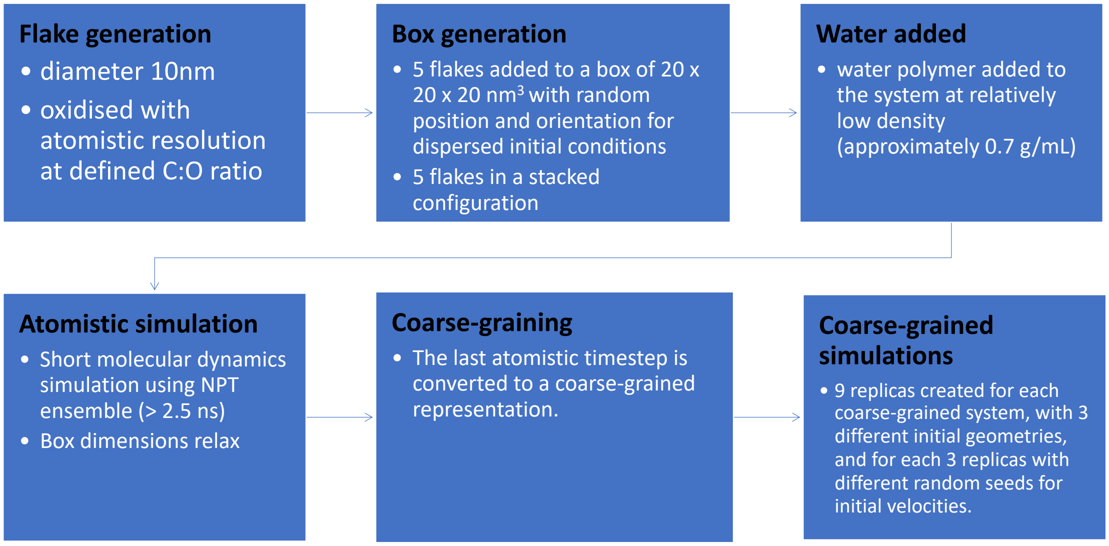

Figure S11: A schematic diagram illustrating the workflow used to create the coarse-grained aqueous GO systems described in the main paper.

To add the water to the simulation box we randomly added water molecules to the simulation box up to the required density, again ensuring no water molecule overlaps with another water molecule or with the added GO flakes.

These atomistic aqueous graphene (oxide) systems were simulated using the OPLS force-

field for the GO flakes and TIP4P/2005 for water, for between 2.5 - 5.0 ns, using the LAMMPS molecular dynamics code. An NpT ensemble was used, at a temperature of 300K and 1atm pressure. The SHAKE constraint algorithm was used to fix the length of the O-H and C-H bonds. The timestep used was 2.0fs. The lattice parameters were relaxed, and the final atom positions were used to generate the CG representation, using the mapping operator described above.

For each system, 9 replicas were created, which consisted of 3 different initial starting configurations, and of these there are three replicas each with differing initial seeds to the CG atom velocities (drawn from a Maxwell-Boltzmann distribution). All CG simulations were performed using a simulated annealing routine to overcome energy barriers. The simulations were performed at 500K for at least 100ns, and cooled down to 300K over 30ns. All subsequent analysis is performed on the configurations generated at 300K. All simulations used the LAMMPS molecular dynamics code, using a RESPA multi-timestepping integrator, with 5.0fs for the non-bonded interactions, and 1.25fs for the bonded interactions. In Table S2, we list the size and total number of atoms of the graphene oxide systems. This size of simulation, and the number of simulations, is unprecedented for studying the behaviour of graphene-oxide systems in water. Each atomistic system contained approximately 590,000 atoms and the CG systems contained approximately 205,000 CG atoms, and in total 81 simulations were run over 130ns.

In addition to the CG simulations, we have continued a small selection of the simulation runs started at the atomistic level, allowing us to see whether we observe the same GO binding motifs as observed in the CG models. The atomistic models selected were a single replica with a C:O ratio = 2.5, for the uncharged carboxylic acid system, the charged carboxylate ion with Na^+ and the charged carboxylic acid ion with Ca^{2+}. These atomistic simulations were run for 30ns at 500K, and cooled down to 300K over 6ns.

S4 Flake Classifications

The classification of each flake atom into either aggregated or intercalated is given in the main paper. We have calculated the percentage of atoms in a flake as either intercalated or aggregated; these are the dots shown in each graph in Figure 2 in the main paper. In Table S3, we list the mean aggregated and intercalated percentage, averaged over all flakes for that environment (45 flakes, simulated in 9 separate simulations).

S5 Uncharged GO flakes, C:O ratio=2.5

In Figure S12 we show snapshots from the coarse-grained uncharged (*i.e.* low pH), C:O ratio of 2.5 systems. We can see that the GO flakes do not form stacks analogous to graphite, but instead form partially overlapped structures, where not more than three flakes are stacked on top of each other.

S6 Uncharged GO flakes, C:O ratio=10.0

In Figure S13 we show a snapshots from the coarse-grained uncharged (*i.e.* low pH), C:O ratio of 10.0 system. In the selected system from the ensemble, we see the GO flakes have not formed a graphitic-like stack, but have a single flake attached to the side of the stack, interacting in a face-edge binding mode. The remainder of the ensembles all show graphitic-like stacks.

References

(1) Stillinger, F. H.; Weber, T. A. Computer simulation of local order in condensed phases of silicon. *Phys. Rev. B* **1985**, *31*, 5262.

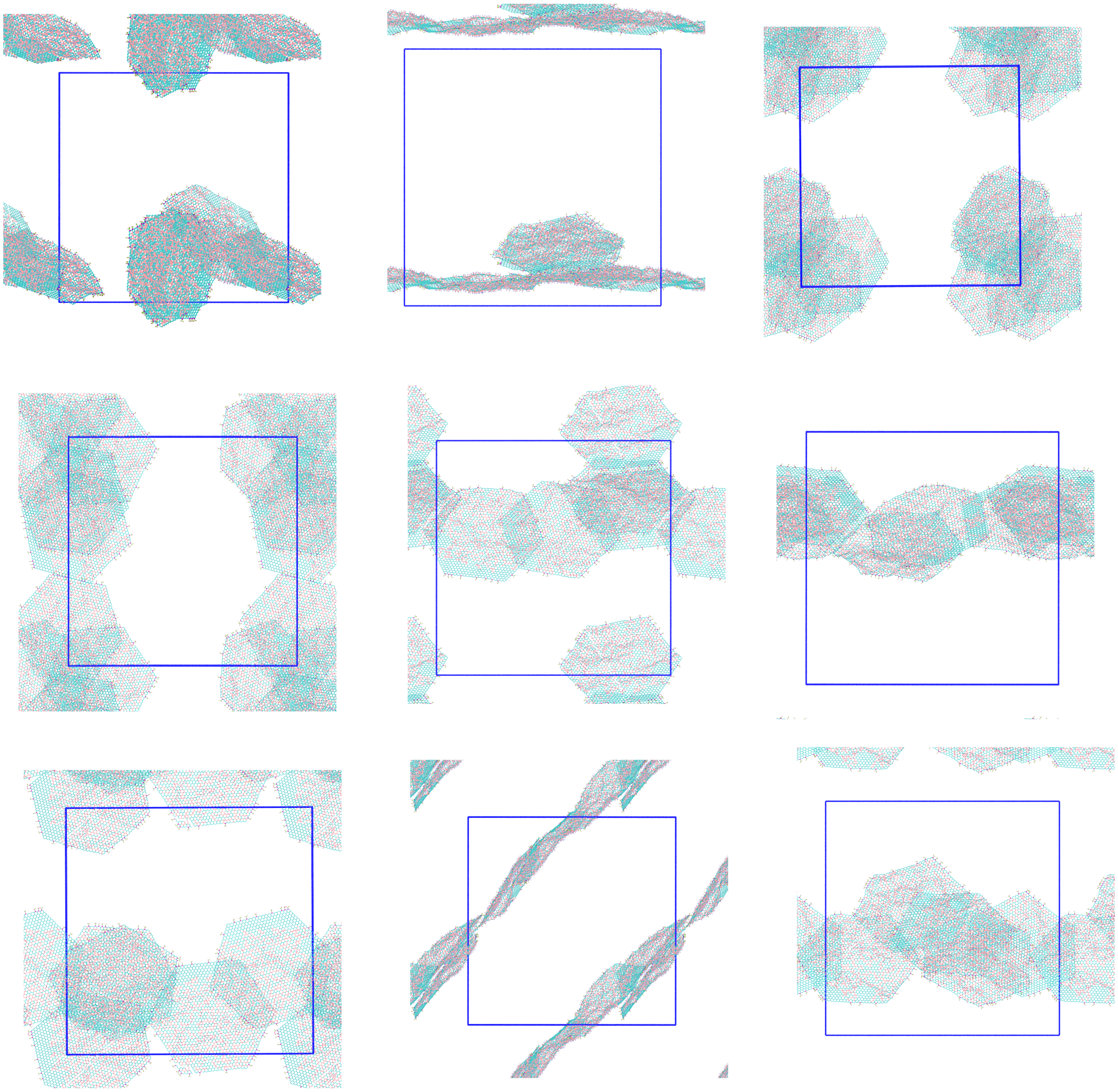

Figure S12: Snapshots from the trajectory of the ensemble of coarse-grained uncharged (*i.e.* low pH), C:O ratio of 2.5 systems. There are nine systems in total. The green represents carbon CG atoms, while red represents oxygen CG atoms. Periodic boundaries are shown as blue lines.

(2) Molinero, V.; Moore, E. B. Water modeled as an intermediate element between carbon and silicon. *J. Phys. Chem. B* **2009**, *113*, 4008–4016.

(3) DeMille, R. C.; Molinero, V. Coarse-grained ions without charges: reproducing the solvation structure of NaCl in water using short-ranged potentials. *J. Chem. Phys.*

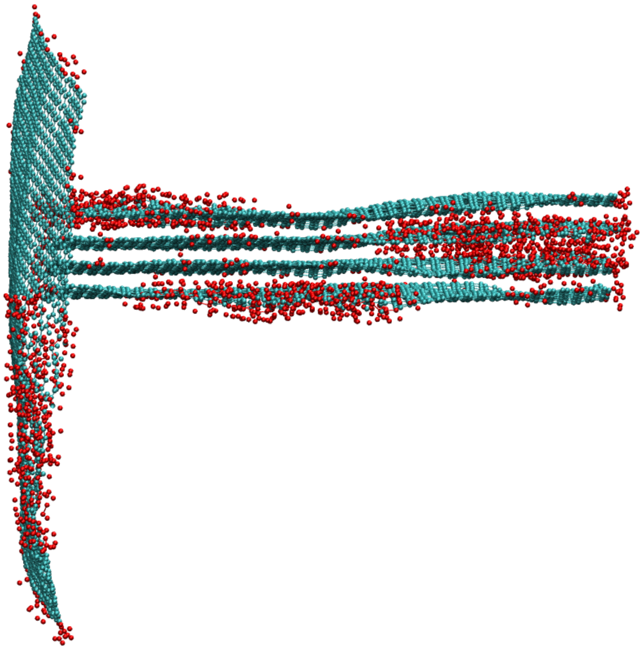

Figure S13: A Snapshots from the trajectory of the ensemble of coarse-grained uncharged (*i.e.* low pH), C:O ratio of 10.0 systems, showing a single flake in a face-edge binding configuration. Green represents carbon CG atoms, red represent oxygen CG atoms.

2009, *131*, 034107.

(4) Gyawali, G.; Sternfield, S.; Kumar, R.; Rick, S. W. Coarse-grained models of aqueous and pure liquid alkanes. *J. Chem. Theory Comput.* **2017**, *13*, 3846–3853.

(5) Islam, N. N.; Sharma, A.; Gyawali, G.; Kumar, R.; Rick, S. W. Coarse-grained models for constant pH simulations of carboxylic acids. *J. Chem. Theory Comput.* **2019**, *15*, 4623–4631.

(6) Suter, J. L.; Sinclair, R. C.; Coveney, P. V. Principles governing control of aggregation and dispersion of graphene and graphene oxide in polymer melts. *Adv. Mater.* **2020**, *32*, 2003213.

Table S2: The aqueous graphene-oxide models studied in this paper. For each system, 9 replica simulations were run from 3 different initial conditions and with different initial seeds to the velocities randomly drawn from a Maxwell-Boltzmann distribution.

C:O ratio	edge group conditions	counterion	box dimensions	number of atoms	number of CG atoms
10.0	carboxylic acid		180.2 Å^3	584825	203375
5.0	carboxylic acid		180.3 Å^3	587265	204535
2.5	carboxylic acid		180.5 Å^3	592040	206980
10.0	carboxylate ion	Na	180.2 Å^3	584885	203530
5.0	carboxylate ion	Na	180.2 Å^3	587330	204665
2.5	carboxylate ion	Na	178.3 Å^3	592175	207150
10.0	carboxylate ion	Ca	180.1 Å^3	584805	203440
5.0	carboxylate ion	Ca	179.4 Å^3	587210	204680
2.5	carboxylate ion	Ca	179.5 Å^3	592095	207100

Table S3: Mean and standard deviation of the final aggregated and intercalated and assembled values for every flake environment. Each row represents the average of 45 flakes simulated in 9 separate simulations of each environment.

C:O ratio	edge group conditions	counterion	aggregated		intercalated	
			mean	std	mean	std
10.0	carboxylic acid		0.97	0.15	0.01	0.05
5.0	carboxylic acid		0.98	0.07	0.01	0.05
2.5	carboxylic acid		0.75	0.20	0.15	0.09
10.0	carboxylate ion	Na	0.76	0.38	0.12	0.17
5.0	carboxylate ion	Na	0.76	0.36	0.13	0.18
2.5	carboxylate ion	Na	0.47	0.31	0.29	0.14
10.0	carboxylate ion	Ca	0.58	0.49	0.03	0.07
5.0	carboxylate ion	Ca	0.30	0.45	0.02	0.06
2.5	carboxylate ion	Ca	0.40	0.39	0.11	0.10

(7) King, M.; Pasler, S.; Peter, C. Coarse-grained simulation of CaCO3 aggregation and crystallization made possible by nonbonded three-body interactions. *J. Phys. Chem. C* **2019**, *123*, 3152–3160.

(8) Souaille, M.; Roux, B. Extension to the weighted histogram analysis method: combining umbrella sampling with free energy calculations. *Comput. Phys. Commun.* **2001**, *135*, 40–57.

(9) Sinclair, R. C. make-graphitics. `https://github.com/velocirobbie/`

make-graphitics, 2020; Accessed: 2021-06-05.

(10) Sinclair, R. C.; Coveney, P. V. Modelling nanostructure in graphene oxide: inhomogeneity and the percolation threshold. *J. Chem. Inf. Model.* **2019**, *59*, 2741.

(11) Sinclair, R. C. make-graphitics. 2019; https://doi.org/10.5281/zenodo.2548538, 10.5281/zenodo.2548538.

(12) Lerf, A.; He, H.; Forster, M.; Klinowski, J. Structure of Graphite Oxide Revisited. *J. Phys. Chem. B* **1998**, *102*, 4477–4482.

(13) Yang, J.; Shi, G.; Tu, Y.; Fang, H. High Correlation between Oxidation Loci on Graphene Oxide. *Angew. Chem. Int. Ed. Engl.* **2014**, *53*, 10190–10194.
